# Bacterial cell-free DNA profiling reveals the co-elevation of multiple bacteria in newborn foals with suspected sepsis

**DOI:** 10.1016/j.isci.2025.114005

**Published:** 2025-11-11

**Authors:** Li-Ting Chen, Emmy Wesdorp, Myrthe Jager, Esther W. Siegers, Mathijs J.P. Theelen, Nicolle Besselink, Carlo Vermeulen, Aldert L. Zomer, Els M. Broens, Jaap A. Wagenaar, Jeroen de Ridder

**Affiliations:** 1 Center for Molecular Medicine, University Medical Center Utrecht, Utrecht University, Utrecht 3584 CX, the Netherlands; 2 Oncode Institute, Utrecht 3521 AL, the Netherlands; 3 Department of Clinical Sciences, Faculty of Veterinary Medicine, Utrecht University, Utrecht 3584 CM, the Netherlands; 4 Department of Biomolecular Health Sciences, Faculty of Veterinary Medicine, Utrecht University, Utrecht 3584 CL, the Netherlands; 5 Wageningen Bioveterinary Research, Lelystad 8221 RA, the Netherlands

**Keywords:** Equine microbiology, Equine pediatric medicine, Microbial genomics

## Abstract

Sepsis is the leading cause of death in neonatal foals, yet current diagnostics lack sufficient sensitivity and specificity. Here, we present a foal cell-free DNA (cfDNA) sequencing for bacterial identification (cfFBI) workflow that integrates wet-lab and computational protocols, enabling direct bacterial profiling through enrichment of the bacterial cfDNA and minimization of false-positive detections. We applied cfFBI to blood from 25 hospitalized foals and 7 healthy foals (H). Sepsis-associated bacterial genera were elevated in all 11 nSIRS-positive (S+) foals compared to H, and in 8/11 when compared to both nSIRS-negative (nS-) and H, with multiple genera elevated in nearly half (45.5%). While total cfDNA concentration, bacterial fraction, and microbial diversity did not differ between groups, S+ foals showed distinct cfDNA end-motif patterns and reduced mitochondrial cfDNA fractions. These findings indicate that cfDNA sequencing enables the detection of pathogenic bacteria and can help identify additional (host-related) sepsis biomarkers.

## Highlights

• Established cfFBI, workflow for bacterial profiling from cfDNA of foal liquid biopsies
• All 11 nSIRS-positive foals had elevated cfDNA of sepsis-associated bacterial genera
• Co-elevation of multiple bacterial genera was seen in 5 of 11 nSIRS-positive foals
• nSIRS-positive foals had host cfDNA with distinct end-motifs and low mt-cfDNA

## Graphical abstract

**Figure:**
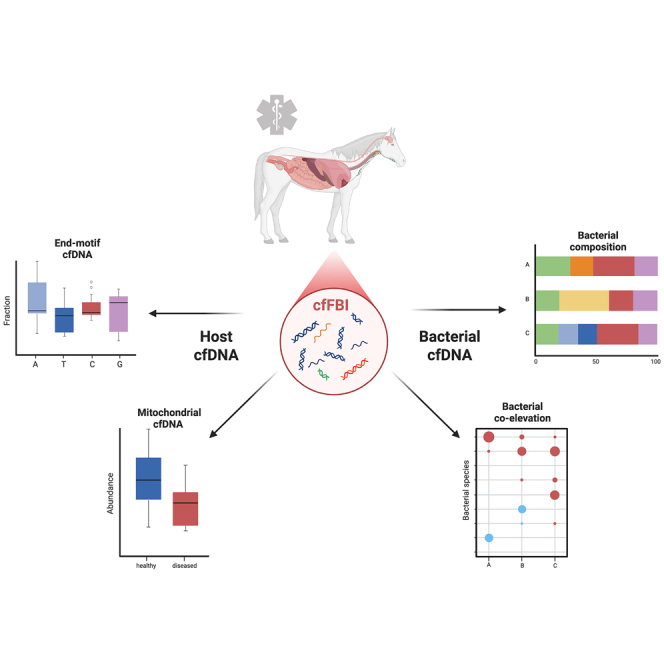

## Introduction

Sepsis is defined as “a life-threatening organ dysfunction caused by a dysregulated host response to infection,” hallmarked by the systemic inflammatory response syndrome (SIRS) and often caused by a bacterial infection.^1^^,^^2^ SIRS arises when pathogen- and damage-associated molecular patterns (PAMPs and DAMPs), as well as neutrophil extracellular traps (NETs), are recognized by the immune system.^3^^,^^4^ Dysregulated innate and adaptive immune responses, in combination with the overactivation of the coagulation system, can result in multiple organ dysfunction, followed by multiple organ failure, and ultimately result in death.^5^^,^^6^^,^^7^ In newborn foals, sepsis stemming from a bacterial infection is an important cause of morbidity and mortality during the first week of life.^8^^,^^9^^,^^10^ Due to the rapid progression of sepsis, early recognition, prompt identification of the causative bacterial pathogen, and timely initiation of effective antimicrobial therapy are critical for improving survival rates.^11^

Despite being a common cause of death in newborn foals, knowledge gaps persist regarding the pathogenesis, diagnosis, and treatment of sepsis. For instance, multiple bacteria are known to co-occur in 2–14% human patients with sepsis (i.e., polymicrobial sepsis),^12^^,^^13^^,^^14^ and a similar phenomenon is likely in newborn foals.^11^^,^^15^ However, traditional culture methods often lack the sensitivity to detect such co-infections. Additionally, when multiple bacteria are cultured from a single sample, it is frequently dismissed as contamination in clinical settings. A further complication in understanding sepsis pathogenesis and diagnostics is that newborn foals absorb immunoglobulins from the colostrum over the gastrointestinal barrier, during which bacteria (including those that can cause sepsis) can also enter the bloodstream.^16^ Although transient bacteremia is a normal physiological process, it remains unclear why, in some foals, this might lead to SIRS and sepsis, while in the majority, it does not.

Significant variability in definitions for equine neonatal sepsis exists in research and a consensus on the criteria that should be used to classify a foal as septic is currently lacking.^2^ To aid the prompt identification of foals at risk of sepsis, several scoring systems have been developed. The systemic inflammatory response syndrome (SIRS) criteria and the neonatal SIRS (nSIRS) criteria are two systems often used to identify foals with suspected sepsis in a clinical setting. These systems use either four (SIRS) or six (nSIRS) objective clinical criteria (Table S1).^2^ These SIRS criteria are based on the human SIRS criteria; abnormal body temperature, tachycardia, tachypnea, and abnormal white blood cell count,^17^ and are modified for use in equids. In human medicine, the need for specific SIRS criteria for use in pediatric patients was recognized, and these were published in 2005.^18^ In accordance with this, in 2015, the equine SIRS criteria were expanded with two additional neonatal SIRS criteria, blood lactate and blood glucose, for use in foals.^19^ Both parameters are associated with sepsis in foals.^20^^,^^21^ Despite the significant work that has been done to evaluate the different scoring systems, it should be recognized that these systems have significant limitations. They have limited sensitivity (SIRS 60%; nSIRS 42%) and specificity (SIRS 69%; nSIRS 76%) for detecting neonatal sepsis.^2^ Bacterial infection, the other hallmark of sepsis, is typically identified through blood cultures, which enable bacteriological identification and subsequent antimicrobial susceptibility profiling. However, the sensitivity of bacterial detection through culture is only 25–45% in foals with sepsis.^22^^,^^23^^,^^24^ Quantitative PCR (qPCR) systems have a higher sensitivity (87%),^25^^,^^26^^,^^27^ but are only able to detect a finite set of pathogens, leading to false negative results for pathogens not included in the test. Additionally, false positive results can occur in both culture and qPCR in cases of transient bacteremia or sample contamination.^28^ As a result of the low sensitivity and specificity of current diagnostic tools, many newborn foals with sepsis remain undiagnosed or misdiagnosed. Given that foals can deteriorate rapidly within hours, there is an urgent need for improved diagnostic tools for earlier clinical intervention. Thus, expanding diagnostic capabilities and enhancing our understanding of sepsis-causing bacteria in foals is essential.

Cell-free DNA (cfDNA) consists of short DNA fragments found in body fluids, including plasma, which are released upon cell and microorganism death.^29^ In human medicine, the sequencing of plasma microbial cfDNA shows great promise for detecting bacterial pathogens in conditions including sepsis.^12^^,^^30^^,^^31^ The advantages of cfDNA short-read sequencing include its culture-independent nature, a reasonable turnaround time of 2–3 days (with the potential to speed this to less than one day using an alternative sequencing platform^32^), and the ability to facilitate the unbiased discovery of new pathogens that have not been previously cultured.^30^^,^^31^ High-throughput sequencing of cfDNA may also reveal general differences in microbial composition in plasma associated with disease development.^12^^,^^30^ The abundance and characteristics, such as fragment lengths, fragment end-motifs, and mapping locations of cfDNA molecules, can reveal information on physiological and pathological processes such as the immune response.^33^^,^^34^^,^^35^ In human plasma, approximately 99.5% of the cfDNA originates from the host,^36^ which is typically ∼167 bp in length.^37^^,^^38^ Microbial cfDNA is shorter, with a substantial fraction being smaller than 100 bp in plasma.^39^^,^^40^^,^^41^ Taxonomic classification and quantification of the microbial cfDNA provides a multi-pathogen, minimally invasive, accurate assay for diagnosing sepsis in humans,^30^^,^^31^ with cfDNA end-motifs potentially enhancing the overall diagnostic process.^42^^,^^43^

In this study, our primary objective is to assess the potential of blood cfDNA sequencing for detecting elevated cfDNA levels of bacteria associated with sepsis in newborn foals with nSIRS. The secondary objectives are analyzing the overall cfDNA bacterial composition and investigating host cfDNA factors, including their origin and end-motif. While previous research has focused on cfDNA concentrations in septic foals,^44^^,^^45^ sequencing of cfDNA has not been previously conducted, positioning this research as a pioneering effort in the field. This endeavor prompted us to create a specialized foal cfDNA sequencing for bacterial identification (cfFBI) workflow, incorporating both wetlab and open-sourced computational workflows optimized for detecting pathogenic bacteria in foals with suspected sepsis. By applying the cfFBI pipeline to 32 newborn foals, we aim to assess the viability of this approach not only as an alternative diagnostic tool, but also as a method to deepen the understanding of the pathophysiology of nSIRS.

## Results

### Cell-free DNA sequencing in newborn foals with sepsis using cell-free DNA sequencing for bacterial identification

**Figure 1:**
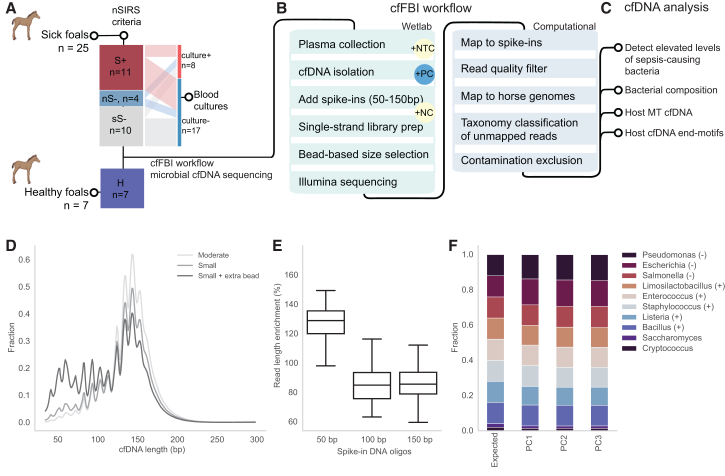
cfFBI pipeline, a cell-free DNA sequencing workflow designed to enhance bacterial identification in foals suspected of sepsis (A) Foal cohort and SIRS categorization: The cohort includes sepsis-suspected foals and healthy (H) controls. Foals were categorized based on nSIRS criteria as SIRS-positive (S+; nSIRS ≥3), SIRS-negative with no symptoms (nS-; nSIRS = 0), or SIRS-negative with symptoms (sS-; nSIRS = 1–2). The alluvial plot shows the number of ill foals with positive blood cultures at hospital admission. (B) Schematic of cfFBI workflow: cfDNA is isolated from foal blood plasma and mixed with synthetic DNA oligos (50, 100, 150 bp). A ligation-based library preparation and bead-based size selection enrich short microbial fragments (<100 bp). After paired-end Illumina sequencing, spike-in sequences and low-quality reads are filtered out. Remaining reads are mapped to host genomes, and unmapped reads are classified taxonomically using Kraken2 with a customized database. Suspected contaminants are finally excluded. The workflow includes diverse controls: positive controls (PC), no-template controls (NTC), and negative controls (NC). (C) Comparative analyses were performed in this study, comparing S+ versus H and nS-. Specifically, we focused on variations in host mitochondrial (MT) cfDNA, chromosomal host cfDNA end-motifs, bacterial load and diversity, and the abundance of potential pathogenic bacteria in septic foals. (D) Comparison of three ligation-based single-strand library preparation methods for enriching short cfDNA fragments: the “moderate small” and “extreme small” protocols from the SRSLY NGS Library Prep Kit, plus an additional bead-based size selection after the “extreme small” protocol. The plot displays the template length size distribution of host cfDNA reads for each method. (E) Enrichment or depletion of synthetic DNA oligos (50, 100, and 150 bp) in foal plasma samples (*n* = 32) is shown in boxplots. Synthetic oligos of 50, 100, and 150 bp were spiked in at equimolar ratios. (F) Taxonomic classification results for a sonicated mock community with 10 microbial species. The symbols (−) and (+) after each bacterial genus name indicate whether the species is Gram-negative or Gram-positive. It compares the expected versus observed species ratios from three technical replicates using the cfFBI workflow and Bracken abundance re-estimation.

To investigate the potential of cfDNA sequencing in the context of equine neonatal sepsis, we prospectively included 25 newborn sick foals admitted to Utrecht University Equine Hospital, as well as seven healthy newborn foals (H) (Figure 1A; Tables S2 and S3). All foals included in this study were between 0 and 6 days of age (Tables S2 and S3). Based on the nSIRS criteria (Table S1),^2^ 11 of these foals were nSIRS-positive (S+; nSIRS≥3), four were nSIRS-negative with zero positive nSIRS parameters (nS-; nSIRS = 0), and 10 were nSIRS-negative but had one or two positive nSIRS parameters (sS-; nSIRS = 1–2) (Figure 1A; Table S3). Three of the eleven S+ (27%), two of the four nS- (50%), and three of the ten sS- (30%) foals had a positive bacterial blood culture (Table S3). Our analyses focused on comparisons between S+ against nS- and/or H foals. sS- foals were evaluated seperately because of the presence of clinical sepsis-related signs (Table S3) suggests that some of these foals could have a bacterial infection or even sepsis. nS- and H foals represent a realistic, clinically relevant background, especially as all nS- samples are derived from the same hospital setting as the S+ samples, ensuring that we account for potential biases related to sample handling and environmental factors.

To enable assessment of elevated levels of pathogenic bacteria in the S+ population, we created a cfFBI wetlab and computational workflow (Figures 1A–1C). Given that the microbial cfDNA fraction is known to be minute in plasma,^39^ cfFBI employs a wetlab strategy to enrich bacterial cfDNA molecules while remaining an untargeted multi-pathogen detection approach. The cfFBI wetlab cfDNA workflow, therefore, consists of a ligation-based single-stranded cfDNA library preparation method followed by a bead-based size selection step, which effectively enriches short (<100 bp) fragments (Figures 1D and 1E), which is the known size range for bacterial cfDNA.^46^

For all foal plasma cfDNA sequencing libraries, between 10 and 50 million paired-end reads were obtained (Figure S1A; Table S4). Since bacterial fractions account for less than 0.5% of the total cfDNA, even after enrichment ^36^, we deemed it crucial to prevent misclassification, especially false positives. cfFBI’s computational pipeline tackles this challenge through a multi-step process designed to minimize such errors. Previous findings demonstrate a reduction of false positive microbial counts by mapping to a more comprehensive host reference genome.^47^ Therefore, cfFBI maps to the latest horse reference genome, EquCab3, along with all other horse genomes available on NCBI, totaling 11 genomes (Figures S2A and S2B), increasing the average host fraction of total cfDNA by 0.5%. Second, cfFBI taxonomically classifies the remaining unmapped reads using Kraken2,^48^ with a custom database that includes all 11 horse genomes, as well as human genomes and all NCBI complete microbial genomes, which improves species assignment. Third, suspected microbial contaminants are excluded from downstream analyses by testing whether the levels of microbial species are correlated with the volume of reagents used in cfFBI (see STAR Methods).^49^

To ascertain the consistency and effectiveness of the cfFBI workflow across library preparations, we first tested the classification accuracy using positive control samples (PCs) from sonicated mock microbial community DNA containing eight bacterial species and two yeasts. In all three technical replicate PCs, the eight bacterial genera were detected at levels consistent with the known microbial composition,^50^ with relative abundance being highly similar across PCs (Figure 1F; Table S5). The consistent detection of the correct microbes in the appropriate ratios across the three different PCs indicates robustness in both the wetlab and the bioinformatics workflow.

### Decontamination and bacterial species composition assessment

We first analyzed the bacterial species composition in the samples, focusing on both the bacterial fraction and its diversity. Typically, 4.2% of the non-mapped reads were confidently classified using Kraken2 (Confidence threshold of 0.8; Figure S1B), of which 1.1% were classified as bacteria at the species level. To ensure accurate analysis of true biological signals, we first removed potential contaminants (see STAR Methods), excluding 0.00319% of bacterial reads classified at the species level that were identified as contaminants (Table S6, Figures S3 and S4). Most of these contaminant species were detected in the negative controls as well (i.e., NTCs and NCs; for details on sample collection, see STAR Methods), indicating that they are likely contaminants from cfDNA isolation or library preparation. The remaining cfDNA reads classified as bacterial species and not identified as contaminants were aggregated for all downstream bacterial composition analyses.

**Figure 2:**
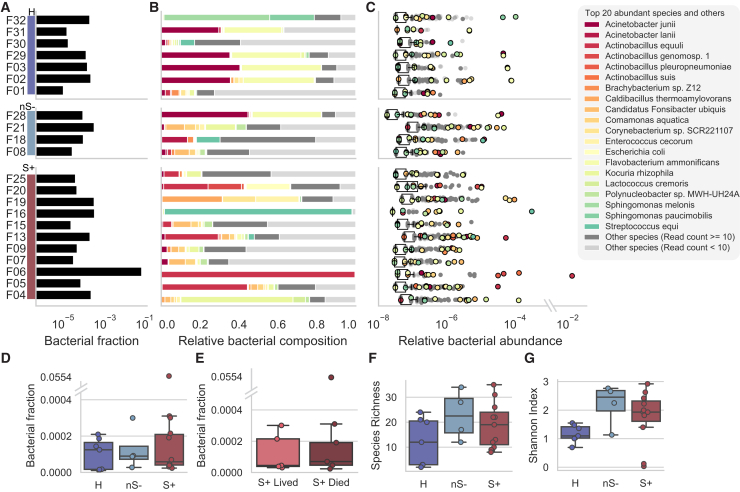
cfDNA bacterial load and diversity in foal plasma samples (A) Bacterial fraction in each sample in three categories H, nS-, and S+, represented in a log scale. (B) Relative bacterial composition (normalized to total bacterial reads) in each sample. The top 20 abundant species in all samples were colored (see colors in the legend), the other species with more than 10 exact counts were colored with dark gray color and the other species with less than 10 exact counts were colored with light gray color. (C) Relative abundance (normalized to total cfDNA reads) in each bacterial species, represented on a log scale. The top 20 abundant species in all samples were colored (see colors in the legend), the other species with more than 10 exact counts were colored with dark gray color and the other species with less than 10 exact counts were colored with light gray. (D) Fraction of cfDNA fragments taxonomically classified as bacterial origin (after removal of contaminant species) and its association with disease status. No significant differences are observed between groups. An outlier at 0.0554 is represented with a broken y axis. Foals in the S+ group showed the largest variation compared to the other two groups. (Standard deviation: H: 0.00008, nS-: 0.00011, S+, 0.0167). (E) Fraction of cfDNA fragments taxonomically classified as the bacterial origin (after removal of contaminant species) and its association with the severity of disease in the S+ group. No significant differences are observed between groups. An outlier at 0.0554 is represented by a broken y axis. (F) Species richness, representing the number of bacterial species identified (after removal of contaminant species) in each plasma sample, and its association with disease status. (G) Shannon index, indicating the evenness of classified bacterial species distribution (after removal of contaminant species) within each foal plasma sample, and the association between Shannon index and disease status. (D–G) Disease status groups are H (*n* = 7), nS- (*n* = 4), and S+ (*n* = 11). For the severity of disease, the focus is on S+ cases with either survival (*n* = 5) or death (*n* = 6). Boxes represent the 25th percentile (bottom), median, and 75th percentile (top), with whiskers extending to the rest of the distribution within 1.5 times the inter-quartile range.

After decontamination, the median bacterial species-classified cfDNA fraction was 0.0083% (range 0.0010–5.5%) (Figure 2A). This total bacterial fraction moderately correlated with age (r = 0.43, Figures S5A and S5B) and was more variable in the S+ group compared to the other two groups, with some samples showing notably high levels, including one outlier at 5.5% (Figures 2A–2D). Although most of the foals with a high bacterial fraction (above 0.0002) were S+ foals (4/6; 66.7%), the difference between groups was not statistically significant (Kruskal-Wallis test with Dunn’s multiple comparison tests) (Figures 2D and 2E). Interestingly, different foals exhibited distinct top abundant species (Figures 2B and 2C). When filtering for species with at least 10 classified reads to account for potential bioinformatics misclassification, between 2 and 37 (median 18.5) species were found across 2 to 30 (median 14) genera. In total, 193 species across 114 genera were detected, with *Actinobacillus*, *Acinetobacter*, *Streptococcus*, and *Flavobacterium* being the most prevalent, contributing a median of 12.3% per foal.

Samples exhibited high variability in species composition (Figures 2B and 2C). The Shannon index revealing greater microbial diversity in sick foals (nS- and S+) compared to healthy foals (Figures 2F and 2G), with age having no significant impact (Figures S5C and S5D). However, neither species richness nor diversity metrics effectively distinguished between healthy, nS-, and S+ foals (Figures 2B, 2C, and S6).

### Co-elevation of multiple sepsis-causing genera observed in foals with sepsis

**Figure 3:**
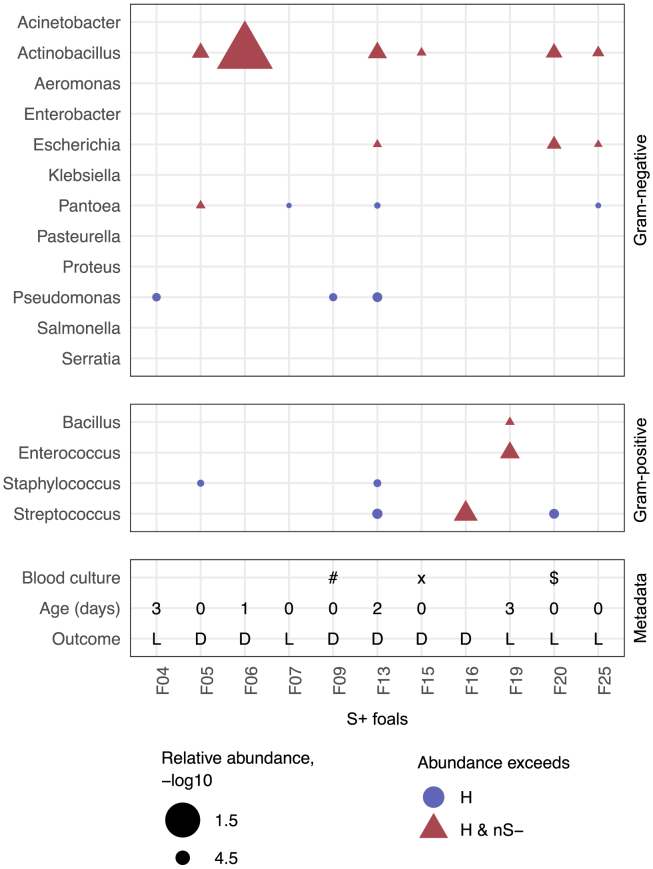
Genus abundance and bacterial co-elevation detection in S+ foal cohort samples Dotplot displays the detection of the 16 most frequently cultured pathogenic genera. Gram-negative species are shown at the top, and Gram-positive species are shown in the middle. Dots represent genera detected with at least 10 reads. Blue circles indicate genera with a relative abundance higher than in H foals, while red triangles denote genera with a relative abundance higher than in both H and nS- foals. Metadata is represented at the bottom, including blood culture if positive, age at hospital presentation if known, as well as survival outcome (L, Lived; D, Died). Special symbols in the blood culture section: “#” Gram-negative rod (non-fermenter), “x” *Actinobacillus equuli* and *Streptococcus pneumoniae*, and “$” *Staphylococcus* coagulase-negative.

The primary objective of this study is to evaluate the potential of blood cfDNA sequencing for detecting elevated levels of sepsis-causing bacteria in newborn foals with nSIRS. To pinpoint bacteria associated with sepsis in the S+ foals, we compared the aggregated counts of species from the 16 most frequently cultured pathogenic genera found in culture-positive foals with sepsis (Figures 3 and S7–S12).^51^ One or multiple pathogenic genera were higher in 8/11 of the S+ foals compared to both nS- and H foals, while the other 3/11 were higher compared to H foals alone (Figure S11), meaning that all S+ foals showed elevated levels of at least one pathogenic genus. In the follow-up analysis, we combined these results for species that showed an increase when compared to either H foals or both nS- and H foals.

Overall, *Actinobacillus* and *Pantoea* were most frequently increased in 6/11 (54.5%) and in 4/11 (36.4%) of the S+ foals, respectively (Figures 3 and S7–S10; Table S7). Co-elevation of multiple genera occurred in 5/11 (45.5%) foals (Figure 3), with the co-elevation of *Actinobacillus* and *Escherichia* being most common (3/11; 27.2%; Figure 3). On the contrary, *Acinetobacter* was the only genus with higher frequencies in multiple H and nS- foals compared to S+ foals (Figure S12), suggesting that the elevated relative abundance of the 16 genera tested represents a genuine biological signal specific to the S+ foals. We did not observe clear relationships between bacterial elevation and the survival outcome of S+ foals (Figure 3), suggesting that survival may be influenced by factors beyond bacterial elevation, including the foal’s immune response and the reaction to treatment. Taken together, these microbial cfDNA sequencing results show that sepsis in foals may have a multi-bacterial nature. Furthermore, the results emphasize that microbial cfDNA sequencing may hold potential for newborn foal sepsis diagnosis, although larger studies are required to establish the sensitivity and specificity of the technique.

Given this promise, we further investigated sS- foals, which were excluded from previous analyses due to the ambiguous disease state. Based on the low sensitivity of the nSIRS criteria (42%) ^2^ and clinical symptoms observed in sS- foals, we expect some foals with sepsis in the sS- group as well. *Acinetobacter* levels were elevated in 4/10 sS- foals compared to S+ (Figure S12), resembling the H foals. Conversely, however, the majority of sS- foals displayed trends similar to S+ foals, including increased levels of *Actinobacillus* (6/10) and *Escherichia* (4/10), when compared to H alone or H and nS-. Additionally, 70% of sS- foals exhibited the co-elevation of multiple genera (Figure S11). The similarities in bacterial co-elevation between sS- and S+ foals, coupled with the low sensitivity of the nSIRS criteria ^2^, suggest that some foals with sepsis may have been overlooked. Alternatively, it could mean that the foals with a low nSIRS score are in an earlier stage of sepsis development or suffer from other bacterial infections, both leading to an increase in microbial levels without many clinical nSIRS symptoms.

Species-level bacterial identification can be used for clinical decision making, including guidance on the selection of antimicrobial treatment. Therefore, we evaluated bacterial species-level elevations in the 16 most common genera associated with foal sepsis. Across the 16 genera, 22 pathogenic species were elevated in one or multiple S+ foals (Table S8, Figures S7–S10). *Actinobacillus equuli*, *Actinobacillus pleuropneumoniae,* and *Escherichia coli* were most frequently elevated in 6/11, 3/11, and 3/11 S+ foals, respectively. Most elevated species corresponded to their respective elevated genera (31/35 observations). However, *Staphylococcus equinus* was higher in F05, *Acinetobacter haemolyticus* was elevated in F04, and *Acinetobacter lanii* as well as *Acinetobacter wanghuae* were elevated in F20, suggesting that species-level information can provide additional insight beyond genus-level analyses (Table S8). However, our analysis also urges caution in interpreting sequencing results, particularly about potential misassignment of reads to closely related species, such as the identification of *Actinobacillus pleuropneumoniae*, which is not typically listed as a sepsis-causing species for foals.

Bacterial culture is the golden standard for identifying bacteria,^24^^,^^28^ but suffers from both low sensitivity and false positive observations.^22^^,^^23^^,^^24^ To evaluate the concordance between traditional culture and bacteria identified (as elevated) by microbial cfDNA profiling through sequencing, we compared blood culture results to cfDNA sequencing. Notably, elevated levels of sepsis-causing bacterial genera were found by cfDNA sequencing in all six foals with positive bacterial blood cultures (three S+ and three sS-), indicating that cfDNA sequencing effectively detects bacterial abnormalities associated with sepsis. In these six foals, eight bacterial isolates were identified by culture with genus-level resolution using MALDI-TOF; six of these were also identified at the species level. Excluding *Staphylococcus equorum* in F20, which is deemed a contaminant during culture, concordance was assessed for the remaining seven bacteria. Of these, 57.1% (4/7) showed elevated levels of cfDNA. In another 28.6% (2/7), cfDNA reads matched the cultured genera or species, but levels were not higher than those in healthy or non-septic nS- foals. This suggests that the bacteria detected by bacterial culture can simply be present rather than elevated in the diseased compared to the healthy setting (Table S9). Taken together, we observe low concordance between culture and cfDNA-based pathogen identification in newborn foals with SIRS, potentially due to the fact that cfDNA sequencing detects the presence and elevated levels of DNA of sepsis-causing bacterial taxa, while culture detects viable bacteria.

### Associations of host cell-free DNA with neonatal systemic inflammatory response syndrome status

**Figure 4:**
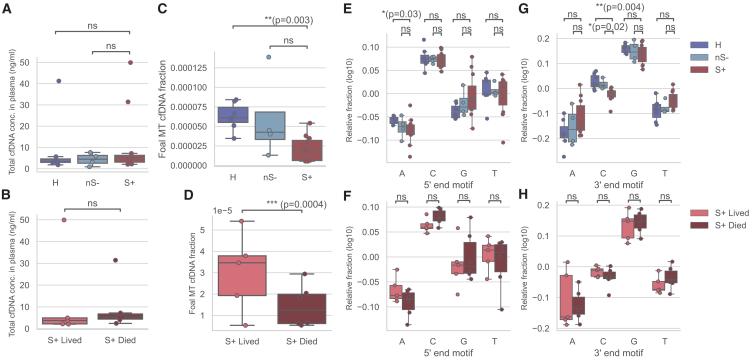
Host cfDNA abundance and its association with disease status and severity of disease (A) Total cfDNA concentration in plasma samples and its association with disease status. ns, not significant (Kruskal-Wallis tests followed by Dunn’s multiple comparison tests. (B) Total cfDNA concentration in plasma samples and its association with severity of disease. ns, not significant (Mann-Whitney U tests). (C) Fraction of cfDNA fragments of host mitochondrial origin, and their association with disease status. ∗∗, *p* < 0.005; ns, not significant (Kruskal-Wallis tests followed by Dunn’s multiple comparison tests. (D) Fraction of cfDNA fragments of host mitochondrial origin and association with severity of disease. ∗∗∗, *p* < 0.001 (Mann-Whitney U tests). (E) Normalized base content fraction at the 5′ end of host chromosomal cfDNA fragments and its correlation with disease status. ∗, *p* < 0.05; ns, not significant (Kruskal-Wallis tests followed by Dunn’s multiple comparison tests. (F) Normalized base content fraction at the 5′ end of host chromosomal cfDNA fragments and its association with severity of disease. ns, not significant (Mann-Whitney U tests). (G) Normalized base content fraction at the 3′ end of host chromosomal cfDNA fragments and its association with disease status. ∗∗, *p* < 0.005; ∗, *p* < 0.05; ns, not significant (Kruskal-Wallis tests followed by Dunn’s multiple comparison tests. (H) Normalized base content fraction at the 3′ end of host chromosomal cfDNA fragments and its association with severity of disease. ns, not significant (Mann-Whitney U tests). Boxes represent the 25th percentile (bottom), median, and 75th percentile (top), with whiskers extending to the rest of the distribution within 1.5 times the interquartile range.

Since most of the sequenced reads are mapped to the host reference genome and it is recognized that these host-derived reads can offer insights into infection related tissue damage,^52^ host response to infection ^29^ and sepsis,^53^^,^^54^ we next investigated differences in host cfDNA between S+ and H and/or nS- foals, and between S+ foals that lived to S+ foals that died. Confirming previous results in foals,^44^ but differing from observations in humans,^53^^,^^54^^,^^55^ total cfDNA levels in plasma were not significantly elevated in S+ foals compared to H and nS- foals (Kruskal-Wallis with Dunn’s multiple comparison; S+ vs. H: *p* = 0.92, Z = 0.73; S+ vs. nS- *p* > 0,99, Z = 0.31), nor in S+ foals that lived compared to S+ foals that died (Mann-Whitney U Test, *p* = 0.32, U = 9, Figures 4A and 4B). This suggests that total cfDNA levels cannot be used to diagnose sepsis in foals, as previously reported,^44^ nor predict disease outcome. Strikingly, opposite to mitochondrial (MT) cfDNA levels in human patients with sepsis,^55^^,^^56^ the MT cfDNA fraction of foal host origin was significantly lower in S+ foals compared to H foals (Figure 4C). Similarly, a significant decrease in MT cfDNA was observed in S+ foals that died compared to those that survived (Figure 4D). Of note, none of these variables were significantly different between isolation batches, library preparation batches, and operators (Mann-Whitney U Test with Bonferroni Correction, Figure S13).

As host end-motifs can give insight into the activities of nucleases and the interplay with innate immune response, such as NETs,^34^^,^^57^ we proceeded to investigate host chromosomal cfDNA end-motifs. An enrichment in 5′ C-end and 3′ G-end cfDNA reads was present in all samples (Figures 4E–4H, Table S10). Specifically, 3′ C-end cfDNA reads were significantly decreased in S+ foals compared to H and nS- foals, while 5′ A-end cfDNA reads were significantly decreased in S+ foals compared to H foals (Kruskal-Wallis test with Dunn’s multiple comparison tests, S+ vs. H: *p* = 0.004, Z = 3.09; Figures 4E–4G, Table S10). Collectively, these results indicate that mitochondrial cfDNA levels and end-motifs could serve as potential biomarkers for SIRS and its prognosis in foals.

## Discussion

We introduce cfFBI, a cell-free DNA sequencing workflow designed to enhance bacterial identification in foals through a combination of optimized wetlab and computational procedures. cfFBI specifically enriches small, microbial cfDNA molecules, which are present at minute levels within the cfDNA pool derived from a single tube of blood. cfFBI’s computational steps, including host mapping and decontamination, are optimized to minimize incorrect bacterial identifications. Using cfFBI, we applied cfDNA sequencing to newborn foals and detected elevated bacterial levels in 8/11 nSIRS-positive (S+) foals compared to levels in (sick) nSIRS-negative (nS-) foals, while all 11/11 nSIRS-positive foals showed at least one bacterial genus elevated compared to healthy foals alone. Interestingly, we found the co-elevation of multiple pathogenic bacteria in 5/11 (45.5%) of nSIRS-positive foals. Although it was already known that bacterial culture can provide positive results for multiple species,^11^^,^^58^ it remained unclear if these were true co-occurrences, similar to the polymicrobial sepsis observed in human patients^12^^,^^13^ or a result of contamination.^23^^,^^24^^,^^58^ The frequent observation of co-elevation in cfDNA in this study suggests that multiple genera may actually jointly contribute to sepsis in newborn foals. Further validation and follow-up research are needed to determine the potential implications of these findings.

Until now, most knowledge about the bacteria causing sepsis in foals has been based on culture-dependent techniques, while culture is known to have only 25–45% sensitivity in foals with sepsis.^22^^,^^23^^,^^24^ When comparing bacteria detection as elevated by cfDNA sequencing to those identified through culture in the cohort, we observed limited concordance (4/7 (57.1%)). Part of this discrepancy may arise because bacterial culture detects only viable bacteria, while cfDNA sequencing reveals both the presence and increased abundance of bacterial cfDNA resulting from recent cell death. The two techniques thereby capture a different aspect of the complex underlying pathophysiology. We argue that it is unlikely that the findings based on cfDNA are false positives since the stringent criteria were chosen for reporting positives and the reported level of this study are on par with previous studies in septic human subjects.^31^^,^^59^^,^^60^ Overall, in large-scale human studies, microbial cfDNA showed higher sensitivity and specificity than blood cultures for detecting clinically relevant pathogens.^61^^,^^62^ The same work showed that including cfDNA in diagnostic workups resulted in enhanced patient survival and reduction of overall antimicrobial use in human patients with sepsis. Ultimately, the two techniques may turn out to be complementary, with cfDNA sequencing providing reliable multi-pathogen results, while culture provides valuable insights into antimicrobial resistance of the identified bacterial species. A promising direction for future research is exploring temporal patterns at bacterial cfDNA levels. Studies in murine sepsis models have shown that serial cfDNA measurements can reveal species-specific dynamics, likely reflecting both its short half-life and the evolving disease state.^63^ Capturing these changes may improve the diagnostic and prognostic utility of cfDNA in veterinary medicine.^63^

Diagnosis of sepsis in newborn foals is challenging, with most tools suffering from low sensitivity and specificity. This issue also applies to the nSIRS scoring system used here, where foals with sepsis can have an nSIRS score of less than three.^2^ To minimize the impact of potential false negative septic foals in the nSIRS-negative group, we excluded the nSIRS- foals with an nSIRS score of one to two when setting the background level for bacterial elevation analysis in nSIRS-positive foals, as some of these cases may have an unindicated sepsis or a bacterial infection. Simultaneously, the nSIRS-positive group may still include foals without sepsis, so without a bacterial infection, as sepsis is currently defined as a combination of SIRS with a bacterial infection. This challenge clearly shows the need for additional diagnostic tools for improved sepsis diagnosis in foals.

Although this study clearly indicates the potential of cfDNA sequencing in newborn foals, it should be noted that the current cohort has limited statistical power to detect significantly elevated microbes in nSIRS-positive foals above background. We opted for a conservative approach to identifying elevated bacterial levels by testing if the value is above the highest observation in the control background samples without aiming to test for significance. A larger cohort study involving more newborn foals is essential to fully assess the potential of cfDNA sequencing for diagnosing sepsis. This would also allow a comparison between healthy and nSIRS-negative foals separately, where the first comparison is informative to gain more insight into biology, whereas the latter comparison can provide tools useful in the clinic. Increased cohort size would also be beneficial to study the abundance of bacterial species or genera in a more unbiased manner, without focusing exclusively on the 16 most frequently observed bacteria in culture. This could provide new insights into the biologically relevant, yet hard-to-culture, bacterial taxa involved in sepsis.

In addition to validating the microbial cfDNA observations of the current study, a larger study could aim to further explore the trends we found in the host cfDNA. This includes confirming the decreased MT cfDNA fraction in nSIRS-positive foals, which can potentially serve as a biomarker. Decreased MT cfDNA fraction is contrary to what is observed in human pediatric sepsis cases,^64^^,^^65^ potentially revealing different disease mechanisms of sepsis in foals and in humans. Moreover, such a study would enable the validation of the absence of the expected elevation in total cfDNA in nSIRS-positive cases,^44^^,^^45^ which would typically be indicative of increased tissue damage and cell death as is seen in humans.^66^ It could also shed light on findings on the (complementary or concordant) relationship between host cfDNA and microbial cfDNA, which we did not observe in the current cohort (Table S10). Finally, the differences in end-motifs in host cfDNA in nSIRS-positive versus nSIRS-negative foals could be validated in a larger cohort, potentially revealing additional biomarkers. By combining host and pathogen information from plasma cfDNA, more insights into host transcription profiles, such as innate immune response activities, can be obtained, as was already shown in human patients with sepsis.^29^^,^^67^

Currently, foals suspected of having sepsis are treated with broad spectrum antimicrobials until culture and susceptibility testing results become available after approximately 72 hours. In cases that fail to improve within this time period, antimicrobial treatment regimens are adjusted based on historical information on the prevalence and susceptibility of bacteria causing sepsis in foals in that specific geographical area. As sepsis and organ dysfunction can develop rapidly, antimicrobial susceptibility tests are rarely timely to aid the treatment regimen. By utilizing a faster and more sensitive technique ^32^ for identifying potential pathogens, coupled with the continually decreasing costs of sequencing combined with more targeted approaches (e.g., for antimicrobial resistance genes^68^), future adjustments to antimicrobial therapy can be made earlier, potentially increasing the survival chances of foals with sepsis.

### Limitations of the study

The current cohort size has limited statistical power to detect genus- or species-level cfDNA elevations in nSIRS-positive foals above the background, since the true distribution of the background level was difficult to determine with only 7 healthy foals and 4 nSIRS-negative foals. The cutoff for detection as elevated was therefore set at the maximum detected level within the SIRS-negative classes (H/nS-). In addition, the current standard diagnostic workflow for sepsis in foals is not always consistent, making it difficult to unequivocally establish if a foal had sepsis or not. Furthermore, potential incomplete representation of equine pathogens in taxonomic databases may yield false negatives or misclassification. Moreover, the detected microbial signals in cfDNA were not corroborated by orthogonal assays such as targeted PCR, which constrains internal validity. Future studies should implement a predefined composite reference standard and use consecutive prospective enrollment to minimize selection and workup biases.^69^^,^^70^

## Resource availability

### Lead contact

Further information and requests for resources and reagents should be directed to and will be fulfilled by the lead contact, Jeroen de Ridder (j.deridder-4@umcutrecht.nl).

### Materials availability

The biological reagents used in this study are available from the lead contact upon request.

### Data and code availability

• Data availability - Metagenomic sequencing data (FASTQ files) have been deposited in the European Nucleotide Archive (ENA) Browser under accession PRJEB77374. The submitted files are labeled using the same Foal ID (FID) as provided in Table 3, following the pattern {FID}_R[1/2].fastq.gz.
• Code availability - The code related to the analysis and visualization of content in this article is deposited at a GitHub repository: https://github.com/AEWesdorp/cfFBI. The repository is open access with a GNU general public license version 3.
• There are no additional resources beyond the data and code reported in this section. For additional information, please contact the corresponding author.

## Acknowledgments

This study is supported by the funding of Stichting Vrienden Diergeneeskunde, the Faculty of Veterinary Medicine, Utrecht University, and a Vidi Fellowship (639.072.715) to JdR from the Dutch Research Council (10.13039/501100003246Nederlandse Organisatie voor Wetenschappelijk Onderzoek, NWO). We thank Rhoxane Korthals from Dierenkliniek Emmeloord for her assistance with sample collection. We thank Joost van Rosmalen for his valuable input on statistical analysis. We acknowledge the Utrecht Sequencing Facility (USEQ) for providing sequencing services and data. USEQ is subsidized by the University Medical Center Utrecht and The Netherlands X-omics Initiative (NWO project 184.034.019). We thank Marc Pagès-Gallego and Dieter Stoker for proofreading the article.

## Author contributions

L.C., E.W., M.J., E.S., M.T., C.V., J.d.R. conceptually designed the study. E.S. and M.T. collected samples, gathered clinical information, and gave clinical input. E.W., E.S., N.B., C.V., M.T. prepared samples. E.W., N.B. and C.V. optimized protocols and generated sequencing libraries. L.C., E.W., M.J. contributed to data analysis. C.V., A.Z., E.B., J.W. provided input on the experiments and analyses. L.C., E.W., M.J. wrote the article. J.d.R. coordinated the study. All authors have read and approved the final article.

## Declaration of interests

JdR is founder and shareholder of Cyclomics BV, a genomics company. The other authors declare no competing interests.

## Declaration of generative AI and AI-assisted technologies in the writing process

During the preparation of this work the authors used chatGPT in order to rephrase sentences. Following its use, the authors reviewed and edited the content as needed and take full responsibility for the content of the published article.

## STAR★Methods

### Key resources table

**Table:** 

REAGENT or RESOURCE	SOURCE	IDENTIFIER
**Biological samples**		

Aseptically collected blood sample from the jugular or cephalic vein	This paper	–
Nuclease Free water	Invitrogen	Cat# 10977-035

**Chemicals, peptides, and recombinant proteins**		

ZymoBIOMICS Microbial Community DNA Standard	Zymo Research	D6305

**Critical commercial assays**		

Circulating Nucleic Acid Kit	Qiagen	Cat# 55114
SRSLY PicoPlus NGS Library Prep Kit	Claret BioScience	CBS-K250B-96
Ampure XP Beads for DNA Cleanup	Beckman Coulter	A63882
Qubit dsDNA High Sensitivity Assay Kit	Thermofisher Scientific	Q32854
Qubit dsDNA Broad Range Assay Kit	Thermofisher Scientific	Q32853
Tapestation D1000 kit	Agilent	Cat# 5067-5583

**Deposited data**		

Healthy and diseased foal cell-free DNA whole-genome sequencing data	European Nucleotide Archive Browser (ENA)	PRJEB77374

**Oligonucleotides**		

50bp oligo: NNNNNNNCGACACGGA TATTCCATCAAGAGACGGGCCTAT GGTCCCTGTGATGATGTNNNNNNN	IDT DNA	–
100bp oligo: NNNNNNNGTAAATCCCA CACAGCTGTCGGCTTATATGGTCATT GGACGGCGTAATAGACAAGAGGA GCATCCGTATTACCGCCTATATCGC CTACGTTTAGAGCATTNNNNNNN	IDT DNA	–
150bp oligo: NNNNNNNGCTCTGGTC AGCCTCTAATGGCTCGTAAGATAG TGCAGCCGCTGGTGATCACTCGAT GACCTCGGCTCCCCATTGCTACTAC GGCGATTCTTGGAGAGCCAGCTGCG TTCGCTAATGTGAGGACAGTGTAGTA TTAGCAAACGATAAGTCNNNNNNN	IDT DNA	–

**Software and algorithms**		

Python v3.10	Python Software Foundation	https://www.python.org
R	R Foundation	https://cran.r-project.org
Prism v10.3.0	GraphPad	https://www.graphpad.com/
Adobe illustrator v28.6	Adobe	–
BioRender 2024	BioRender	https://www.biorender.com/
Customized code	N/A	https://github.com/AEWesdorp/cfFBI

**Other**		

MALDI-TOF	Bruker	–
Tapestation 2200	Agilent	–
Streck Cell-Free BCT	Streck	Cat# 230257
Covaris S2	Covaris	–
NovaSeq 6000	Illumina	–

### Experimental model and study participant details

#### Foal cohort

We prospectively included 25 sick foals admitted to the Utrecht University Equine Hospital (Utrecht, The Netherlands), between March 1st, 2021 and July 1st, 2022. For diagnostic purposes, two blood samples (up to 20 mL each) and one blood sample of 10 mL were collected aseptically from the jugular or cephalic vein, either by venepuncture or through a newly placed intravenous catheter immediately upon hospitalization. The two 20 mL samples were placed into 70 mL brain heart infusion broth + SPS (Biotrading, Mijdrecht, the Netherlands) and transported to the Veterinary Microbiological Diagnostic Center where the bottles were incubated at 37°C for 18-24h. After incubation, Gram-staining was performed followed by inoculation on two sheep blood agars (SBA), chocolate agar (CHOC) and MacConkey agar (MAC; Biotrading, Mijdrecht, the Netherlands). One SBA and MAC agar were incubated aerobically, while the other SBA was incubated anaerobically and the CHOC agar was incubated microaerobically; all at 37°C for 5–7 days. Agars and broths were checked daily for bacterial growth. If bacterial growth was detected, identification took place using Maldi-TOF (Bruker, Bremen, Germany). The 10 mL sample of blood was collected directly into a Streck tube (see “sample preparation and nucleic acid isolation”). Bacteriology culture results were recorded at the species level when Maldi-TOF reported a high confident score, whereas a genus level or a higher taxon level result was recorded when Maldi-TOF reported intermediate or low scores. Diagnostic and clinical data of sick foals were recorded and later extracted from the medical information system (Table S3).

In addition to the sick foals, seven healthy (H) newborn foals were enrolled in this study, four from Utrecht University Equine Hospital (Utrecht, The Netherlands) and three from Dierenkliniek Emmeloord (Emmeloord, The Netherlands). In the healthy foals, at the moment of blood collection for the routine check for passive transfer of immunity, 10 mL of blood was collected aseptically for cfDNA sequencing. Blood cultures were not performed on the healthy foal samples.

nSIRS criteria were used to classify foals into groups (Table S1) ^2^, whereby an nSIRS score ≥3 was considered nSIRS-positive (S+), a nSIRS score of 0 was considered nSIRS-negative (nS-) and an nSIRS score of 1–2 was considered symptomatic nSIRS-negative (sS-). The foal cohort thus comprised 11 S+ foals, four nS- foals, seven healthy foals, and 10 sS- foals. The demographics, including foal age at presentation, breed, sex, as well as dam age, gestation length, and parity, are detailed in Tables S2 and S3.

### Method details

#### Sample preparation and nucleic acid isolation

Blood for cfDNA sequencing was collected aseptically in Streck Cell-Free BCT (Streck #230257). Plasma extraction involved centrifugation for 10 min at 1600 g (at room temperature), followed by an additional centrifugation step for 10 min at 16,000 g (at 4 °C) to eliminate all cells and debris. The resulting plasma samples were then stored at −80 °C.

For nucleic acid isolation from plasma, the Circulating Nucleic Acid Kit (Qiagen, 55114) was employed with specific modifications to the manufacturer’s protocol. First, a subset of the samples was supplemented to 5 mL using PBS (Table S4), prior to isolation. Second, the lysis time was extended from 30 to 60 min. Finally, cfDNA was eluted in 28 or 35 μL of Nuclease Free water (Invitrogen, 10977-035), and measured by the Qubit dsDNA High Sensitivity Assay Kit or Broad Range Assay Kit (Thermofisher Scientific, Q32854 and Q32853, respectively).

#### Sequencing library preparation using single-strand ligation based DNA-capture

For library preparation quality control purposes, plasma DNA was supplemented with synthetic spike-ins, equaling 0.2% of the total DNA input. The synthetic spike-ins consisted of an equimolar mix of three single-stranded DNA sequences that were 50, 100, and 150 bp in length (sequences of these spike-ins listed in Table S11). Since the SRSLY splint adapter (refer to the next section for more details about SRSLY) contains a 7-base random overhang, the spike-ins were designed to include a random overhang sequence of the same length.

Then, the SRSLY PicoPlus NGS Library Prep Kit was used to prepare sequencing libraries (Claret BioScience, CBS-K250B-96). Briefly, DNA input molecules were denatured and kept as single-stranded molecules using a thermostable single-stranded DNA binding protein. The single-stranded DNA was then ligated to SRSLY splint adapters, followed by an indexing PCR.^71^ To enrich short fragments in all foal samples, we used the small fragment retention version of the SRSLY PicoPlus NGS Library Prep Kit protocol along with an additional bead-based size selection step (Ampure XP, A63882). In a separate experiment (Figure 1D) we compared a short fragment retention and moderate fragment retention protocol combined with/without a customized extra step of bead-based selection in a separate experiment, by which we established that we would use the small fragment retention version with a customized extra step of bead-based selection in all other samples in this study. The complete description of this experiment can be found in the method section “short fragmentation length enrichment analysis”.

All libraries were quantified using the Qubit dsDNA High Sensitivity Assay Kit (Thermofisher Scientific, 32854) and size distribution was analyzed using the Tapestation 2200 and the D1000 kits (Agilent, 5067–5583). Foal sample sequencing libraries were pooled equimolar, with positive (see “preparing positive control samples imitating microbial cfDNA fragments”) and negative controls (see “negative controls for identifying contaminants in low microbial load samples”), albeit at a 3-fold lower molar ratio than the foal cfDNA libraries. This pool was subsequently enriched for sub-100 bp cfDNA molecules by a bead-based size selection step (Ampure XP, A63882). After this bead-based size selection, the concentration and size of the library pool were measured using the TapeStation 2200 and the D1000 kit.

#### Preparing positive control samples imitating microbial cfDNA fragments

As a positive control, we made use of a sonicated mock community DNA (ZymoBIOMICS Microbial Community DNA Standard, D6305) containing a mixture of genomic DNA of 10 microbial strains: *Listeria monocytogenes, Pseudomonas aeruginosa, Escherichia coli, Salmonella enterica, Lactobacillus fermentum, Enterococcus faecalis, Staphylococcus aureus, Bacillus subtilis, Saccharomyces cerevisiae* and *Cryptococcus neoformans*. In short, 2 μL ZymoBIOMICS standard was supplemented with 88 μL of LowTE (10 mM Tris, 0.1 mM EDTA), before shearing using the Covaris S2 at 6°C–8°C, with continuous degassing, a duty cycle of 10%, intensity set to 5, and 200 cycles per burst for 14 min. Bead-based size selection was then performed to enrich for DNA fragments shorter than 200 bp (Ampure XP, A63882), using an initial 1.1x volume of beads followed by adding a 3x volume of beads to the supernatant, to mimic cfDNA. Three ng of sheared, size-selected mock community DNA supplemented with 6 pg of synthetic spike-in DNA were used as input for the SRSLY library preparation. Since the next-generation sequencing libraries were prepared in three separate batches, we included one positive control sample for each batch, resulting in a total of three positive controls (PC1, PC2, and PC3).

After sequencing, the computational cfFBI workflow was applied to the positive control (PC) samples, including Bracken abundance re-estimation to refine the relative fractions of each species and genus (see “sequencing data processing using the cfFBI-pipeline” for details). The observations for these 10 species and their respective genera are provided in this study, including their relative fractions at both the species and genus levels, as well as the variance among the PC samples.

Of note: the positive controls (PC1-PC3) served three purposes. First, to validate the effectiveness of the protocol in each experimental batch. Second, to ensure the wet lab and computational workflow can accurately produce representative species and genera of interest. Third, to confirm that data generated from independent library preparations are comparable.

#### Negative controls for identifying contaminants in low microbial load samples

Due to the risk of contamination in low microbial load samples, we incorporated a set of four negative controls (NTC1, NTC2, NCMQiso, NCMQlib). Among these, two (NTC1, NTC2) consisted of 5 mL PBS that underwent the entire process of cfDNA and SRSLY-mediated NGS sequencing library preparation. Another two control samples contained Nuclease-Free water that was utilized for the elution (NC1MQiso) of cfDNA after cfDNA isolation and the supplemention of up to 18 μl that was added to samples before library preparation (NC1MQlib). These two samples underwent the process of SRSLY library preparation. No spike-in DNA sequences were added to the negative control samples.

#### Next-generation sequencing

Library sequencing was executed on the NovaSeq 6000 platform with 2 x 150 bp reads. This process yielded a range of 20–66 million reads per cfDNA library, between 8.6 and 10.3 million reads for each positive control (PC), and between 6.0 and 9.1 million reads for negative controls (NTC1, NTC2, NC1MQiso, and NC1MQlib).

#### Sequencing data processing using the cfFBI-pipeline

Illumina sequencing and synthetic data underwent processing via the cfFBI-pipeline, available on our Github repository (https://github.com/AEWesdorp/cfFBI/tree/main/pipeline). In a nutshell, bbduk.sh from tool BBmap^72^ was employed to detect and eliminate reads containing 50mer, 100mer, or 150mer synthetic spike-in sequences. Subsequently, duplicate removal was carried out using nubeam-dedup,^73^ followed by default read quality filtering using fastp^74^ to generate high-quality sequencing data. The quality filtering included removing low-quality reads, implementing a low complexity filter, adapter removal, and discarding short reads (<35bp) using AdapterRemoval.^75^

For horse read sequence identification, we tested two strategies via host genome mapping (bowtie2^76^). The first strategy utilized the reference genome *Equus caballus* EquCab3.0 from NCBI RefSeq (accessed on Nov 8th, 2022). The second strategy incorporated all 10 additional genomic sequences available for *Equus caballus* within NCBI RefSeq (accessed on Feb 5th, 2024), bringing the total to 11 genomes: EquCab3.0, 57H, 25H, 16H, 7H, 2H, 9H, 30H, Ajinai1.0, LipY764, and EquCab2.0. A comparison of the two strategies is shown in Figure S2, highlighting a substantial reduction in unmapped host reads (Figure S2A) and the downstream effect on bacterial read classification following remapping with Kraken2 (Figure S2B; see next section for details). To mitigate artificially elevated microbial read counts due to incomplete host filtering, the multi-genome mapping strategy has been adopted in the cfFBI pipeline.

The latter strategy is adopted in the cfFBI pipeline. After host sequence subtraction, remaining paired-end reads underwent taxonomic classification using Kraken2 ^48^, a highly regarded metagenomic tool that performs exact *k*-mer alignment to a reference database for rapid per-read taxonomic classification (for details about the adapted database, see: “taxonomic database construction and taxonomy classification”). Sequencing data were processed with a confidence threshold (CT) of 0.8 for all described databases, the selection of the CT was based on previous work^77^ which demonstrated that a CT of 0.8 results in the highest average precision when using the NCBI database. After Kraken2 classification, Bracken^50^^,^^77^ was employed to re-estimate the abundance of species within the metagenomic PCs (PC1-PC3; as specified in the cfFBI’s config file). Of note, Bracken abundance re-estimation was applied for PCs but not for foal cfDNA samples according to the Kraken software suite authors’ recommendation.^78^ Resulting host-mapping reads, classified reads, and bacterial-classified reads were normalized to QC-passed reads in each sample unless otherwise specified.

#### Taxonomic database construction and taxonomy classification

For this study, we constructed a custom Kraken2 hash-table database that includes 11 horse genomes, two human genomes, and all complete microbial genomes from NCBI (release 217, downloaded as of May 15th, 2023). The microbial component comprises 285,825 bacterial, 14,977 viral, 496 fungal, 1,493 archaeal, and 96 protozoal genome assemblies. To build this database, genomic sequences from the NCBI RefSeq database were downloaded using the *kraken2-build --download-taxonomy* command for archaea, bacteria, fungi, human, plasmid, protozoa, UniVec_Core (contaminant sequences), and viral genomes. Additionally, all 11 genomic sequences for Equus caballus (EquCab3.0, 57H, 25H, 16H, 7H, 2H, 9H, 30H, Ajinai1.0, LipY764, and EquCab2.0) from NCBI RefSeq were downloaded (as of 05-02-2024). The database also incorporated the human genome GRCh38.p14 (obtained directly from NCBI RefSeq) and CHM13v2.0 (added manually). Kraken2 databases were built using the default settings (*kraken2-build*), resulting in a database with k-mers of the default length (35) and minimizers of length 31.

#### Short fragmentation length enrichment analysis

To evaluate the efficiency of short (<100 bp) microbial cfDNA fragment enrichment across various protocols, we tested different versions of the SRSLY PicoPlus NGS Library Prep Kit (Claret BioScience, CBS-K250B-96), including short fragment retention and moderate fragment retention protocol combined with or without a customized extra step of bead-based selection. After preparing these four different libraries, the libraries were pooled and sequenced with NextSeq 2000. On average 29 million paired-end (2 x 150 bp) reads were obtained. The cfFBI computational workflow was applied to all four samples, using only the EquCab3.0 as a reference genome. Reads mapped to the host chromosomal contigs were extracted using samtools (v1.3.1) with a minimal mapping quality score of ≥ 30. Subsequent processing and length analysis were performed by a customized processing script using R (v4.2.1) (for details, see: https://github.com/AEWesdorp/cfFBI/tree/main/fragmentomics).

#### Host mitochondrial and host read end-motifs analysis

Sequences mapped to mitochondrial contig (RefSeq contig name NC_001640.1) and chromosomal contigs of *Equus caballus* EquCab3.0 were extracted for host read mitochondrial and host read end-motif analyses using samtools (v1.19, v1.3.1). Amount of reads mapped to mitochondria with a quality score of ≥ 40 was divided by the number of reads mapped to all chromosomal and mitochondrial DNA with a quality score of ≥ 40 in EquCab3.0 to calculate MT cfDNA fraction. To investigate read end-motifs, we analyzed the most terminal base of R1 and R2 in all reads that were mapped with a quality score of ≥ 30, using a custom R script. Counts of each terminal base were tallied, then normalized to the expected fraction (equally distributed). As an example, for Motif A in 1-mer end-motif:

$$Motif\;A\;relative\;fraction=\log\;10\left(\frac{motifA\;*\;4}{\sum \left(\text{motifA}\;+\;\text{motifB}\;+\;\text{motifC}\;+\;\text{motifD}\right)}\right)$$

#### Contamination identification in sequencing runs with concentration-based methods

Low biomass microbial sequencing is sensitive to any DNA sequence present in samples, including contaminants. Decontamination, which refers to the process of removing contaminants from findings, is crucial to exclude uninformative findings. Previously, “*decontam,*” a statistical method which identifies and removes reagent-related contaminant sequences has been proposed for metagenomics data. This method, implemented in an R package, detects contaminants by analyzing their correlation with DNA concentration (frequency-based method) as well as their presence in negative controls (prevalence-based method) ^49^ (illustration adapted in Figure S3B). We adapted the frequency-based method to identify contaminants resulting from the cfDNA isolation step and/or the SRSLY library preparation step (Figure S3A).

This frequency-based method assumes that contaminants exist at the same concentration in input reagents in different samples. Therefore, contaminants are more abundant in samples with low DNA input and consequently samples with lower DNA yield. We measured the cfDNA yield after isolation and used (whenever possible) 5 ng of cfDNA as input for the library preparation, to standardize the input DNA for this step. As a result, more isolation-related contaminants were expected in lower input DNA concentration samples. After SRSLY sequencing library preparation we again measured the total DNA yield, and pooled samples in equimolar amounts for sequencing, thus, more library preparation-related contaminants were expected in samples with low total DNA yield.

Therefore, both cfDNA isolation yield and SRSLY sequencing library preparation yield were used for contaminant identification. Normalized species classified counts of all species were correlated with (1) the inverse input DNA volume used for the library preparation, against correlation to a constant value (Figures S3C and S3D); and correlated with (2) final DNA yield after library preparation, against correlation to a constant value (Figures S3C and S3E). Testing the null hypothesis of whether each species was not a contaminant from step (1) and/or step (2) with the R package *decontam*. This derived a *p*-value (significance) of the likelihood of being a contaminant for each species. The authors of the decontam package suggested identifying the trough in the distribution of *p*-values to set as a cutoff to identify contaminants. We set a *p*-value cutoff at 0.25 for both steps and checked whether the species occurred in at least six samples, as a lenient approach to identify as many suspected contaminants as possible to prevent false positive findings as suspected pathogens (Figures S3F and S3G). We performed this across all isolation and library preparation samples without batch-specific testing due to small batch sizes in this experiment. The identified contaminant species were removed from the further analysis that involves classification results. To validate that the identified species were true contaminants, we checked their presence in four negative controls (NTC1, NTC2, NC1MQiso, and NC1MQlib, see Figure S4).

#### Bacterial load calculation

After excluding contaminant species, read counts of all other bacterial species were aggregated and normalized to the total number of quality-filtered reads, to calculate what we call the “total fraction of bacterial cfDNA”.

#### Bacterial diversity measurements

For bacterial diversity measurement, it is important to avoid analytical noise arising from potential false positive taxonomy classification. Apart from removing species deemed as contaminants from the above mentioned method, we also exclude all observations that were fewer than ten classified reads. To perform diversity analyses, it is important to rarefy the observation so that it is comparable between libraries. Detected species count were rarefied based on the filtered read count in the smallest sequencing library. Species richness was then calculated as the number of species present in each sample after rarefaction. This includes only species that have more than ten classified reads in any sample and are not identified as contaminant species. The Shannon index, or Shannon entropy, measures biodiversity by considering both the abundance and evenness of species present in a sample.^79^ We also measured Bray-Curtis dissimilarity to assess compositional differences between sample pairs. In order to calculate this Bray-Curtis dissimilarity for all pairs, counts were log10-transformed. Results were visualized using hierarchical clustering with single linkage (nearest neighbor) to illustrate the relationships between samples.

#### Identification elevated bacterial taxa

Using the cfFBI workflow, we aimed to detect elevated levels of frequently observed bacterial pathogens in newborn foals with sepsis ^51^. These bacterial pathogens include 12 Gram-negative genera (*Serratia*, *Salmonella*, *Pseudomonas*, *Proteus*, *Pasteurella*, *Pantoea*, *Klebsiella*, *Escherichia*, *Enterobacter*, *Aeromonas*, *Actinobacillus*, and *Acinetobacter*) and four Gram-positive genera (*Streptococcus*, *Staphylococcus*, *Enterococcus*, and *Bacillus*). We visualized the taxonomy-classified normalized read counts for all species within these 16 genera (Figures S7–S10) and aggregated these counts to form a total count for each genus. Comparisons were made by contrasting the foals’ (e.g., S+) aggregated genus-level counts against the maximum aggregated genus-level counts observed in H and/or nS- foals to identify abundances that exceeded those in the control group (i.e., H and/or nS-). Conversely, we also compared the aggregated genus-level counts of the non-S+ foals against those observed in the S+ group. In all comparisons, genera detected at low abundance (fewer than 10 reads) were excluded.

### Quantification and statistical analysis

We utilized Kruskal-Wallis tests followed by Dunn’s multiple comparison tests to conduct a directional non-parametric ANOVA for comparing total cfDNA levels, mitochondrial cfDNA fraction, host cfDNA end-motifs, bacterial cfDNA fraction, species richness, and Shannon indices among groups composed of H, nS- or S+ foals. For the comparison between two groups (S+ Lived and S+ Died) we used Mann-Whitney U tests. Additionally, batch effects were assessed with Mann-Whitney U tests with Bonferroni correction comparing whether variables of interest were confounded by factors such as the isolation batch, library preparation batch, and the location of the sample during library preparation (Figure S10).

## Supplemental information

Document S1. Figures S1–S13, Tables S1, S2, S8, and Table S11

Table S3. Foal clinical metadata, nSIRS scoring, blood-culture results, and disease outcomes, related to Figure 1

Table S4. Per-sample library-preparation metadata, along with sequencing and mapping summary, related Figure 2

Table S5. Positive-control mock community composition and recovery metrics across three libraries (PC1–PC3), related to Figure 2

Table S6. Species-level contaminants attributed to cfDNA isolation or library preparation who are identified by decontam49, related to Figure 2

Table S7. Genus-level signals of sepsis-associated bacteria across all foals, stratified by Gram status and clinical class, related to Figure 3

Table S9. Concordance between positive blood culture and cfDNA metagenomic profiling at the genus and species level, related to Figure 3

Table S10. Overview of clinical outcome, total cfDNA, mitochondrial cfDNA, bacterial cfDNA and host cfDNA end-motif enrichment per foal grouped by disease status, related to Figure 4

## References

[1] Singer M., Deutschman C.S., Seymour C.W., Shankar-Hari M., Annane D., Bauer M., Bellomo R., Bernard G.R., Chiche J.-D., Coopersmith C.M. (2016). The Third International Consensus Definitions for Sepsis and Septic Shock (Sepsis-3). JAMA.

[2] Wong D.M., Ruby R.E., Dembek K.A., Barr B.S., Reuss S.M., Magdesian K.G., Olsen E., Burns T., Slovis N.M., Wilkins P.A. (2018). Evaluation of updated sepsis scoring systems and systemic inflammatory response syndrome criteria and their association with sepsis in equine neonates. J. Vet. Intern. Med..

[3] Cicchinelli S., Pignataro G., Gemma S., Piccioni A., Picozzi D., Ojetti V., Franceschi F., Candelli M. (2024). PAMPs and DAMPs in Sepsis: A Review of Their Molecular Features and Potential Clinical Implications. Int. J. Mol. Sci..

[4] Denning N.-L., Aziz M., Gurien S.D., Wang P. (2019). DAMPs and NETs in Sepsis. Front. Immunol..

[5] Dunkel B., Corley K.T.T. (2015). Pathophysiology, diagnosis and treatment of neonatal sepsis. Equine Vet. Educ..

[6] Eaton S. (2023). Neonatal sepsis – Pathology and clinical signs. Equine Vet. Educ. Equine Vet. Educ..

[7] Sheats M.K. (2019). A Comparative Review of Equine SIRS, Sepsis, and Neutrophils. Front. Vet. Sci..

[8] Cohen N.D. (1994). Causes of and farm management factors associated with disease and death in foals. J. Am. Vet. Med. Assoc..

[9] Wohlfender F.D., Barrelet F.E., Doherr M.G., Straub R., Meier H.P. (2009). Diseases in neonatal foals. Part 1: the 30 day incidence of disease and the effect of prophylactic antimicrobial drug treatment during the first three days post partum. Equine Vet. J..

[10] Galvin N., Corley K. (2010). Causes of disease and death from birth to 12 months of age in the Thoroughbred horse in Ireland. Ir. Vet. J..

[11] Theelen M.J.P., Wilson W.D., Byrne B.A., Edman J.M., Kass P.H., Magdesian K.G. (2019). Initial antimicrobial treatment of foals with sepsis: Do our choices make a difference?. Vet. J..

[12] Chen P., Li S., Li W., Ren J., Sun F., Liu R., Zhou X.J. (2020). Rapid diagnosis and comprehensive bacteria profiling of sepsis based on cell-free DNA. J. Transl. Med..

[13] Doualeh M., Payne M., Litton E., Raby E., Currie A. (2022). Molecular Methodologies for Improved Polymicrobial Sepsis Diagnosis. Int. J. Mol. Sci..

[14] Lee S.Y., Park M.H., Oh D.K., Lim C.-M., Korean Sepsis Alliance (KSA) investigators (2024). Polymicrobial bloodstream infections per se do not increase mortality compared to monomicrobial bloodstream infections in sepsis patients: a Korean nationwide sepsis cohort study. BMC Infect. Dis..

[15] Gayle J.M., Cohen N.D., Chaffin M.K. (1998). Factors Associated with Survival in Septicemic Foals: 65 Cases (1988–1995). J. Vet. Intern. Med..

[16] Perkins G.A., Wagner B. (2015). The development of equine immunity: Current knowledge on immunology in the young horse. Equine Vet. J..

[17] Bone R.C., Balk R.A., Cerra F.B., Dellinger R.P., Fein A.M., Knaus W.A., Schein R.M., Sibbald W.J. (1992). Definitions for sepsis and organ failure and guidelines for the use of innovative therapies in sepsis. The ACCP/SCCM Consensus Conference Committee. American College of Chest Physicians/Society of Critical Care Medicine. Chest.

[18] Goldstein B., Giroir B., Randolph A., International Consensus Conference on Pediatric Sepsis (2005). International pediatric sepsis consensus conference: definitions for sepsis and organ dysfunction in pediatrics. Pediatr. Crit. Care Med..

[19] Wong D.M., Wilkins P.A. (2015). Defining the Systemic Inflammatory Response Syndrome in Equine Neonates. Vet. Clin. North Am. Equine Pract..

[20] Hollis A.R., Furr M.O., Magdesian K.G., Axon J.E., Ludlow V., Boston R.C., Corley K.T.T. (2008). Blood glucose concentrations in critically ill neonatal foals. J. Vet. Intern. Med..

[21] Corley K.T.T., Donaldson L.L., Furr M.O. (2005). Arterial lactate concentration, hospital survival, sepsis and SIRS in critically ill neonatal foals. Equine Vet. J..

[22] Hytychová T. ’ana, Bezděková B. (2015). Retrospective evaluation of blood culture isolates and sepsis survival rate in foals in the Czech Republic: 50 cases (2011-2013). J. Vet. Emerg. Crit. Care.

[23] Russell C.M., Axon J.E., Blishen A., Begg A.P. (2008). Blood culture isolates and antimicrobial sensitivities from 427 critically ill neonatal foals. Aust. Vet. J..

[24] Giancola S., Hart K.A. (2023). Equine blood cultures: Can we do better?. Equine Vet. J..

[25] Poltavchenko G.M. (1990). [Effects of diazepam and N(6)-cyclohexyladenosine on the level of diazepam-binding inhibitor in structures of the hippocampus during immobilization stress]. Biull. Eksp. Biol. Med..

[26] Elmas C.R., Koenig J.B., Bienzle D., Cribb N.C., Cernicchiaro N., Coté N.M., Weese J.S. (2013). Evaluation of a broad range real-time polymerase chain reaction (RT-PCR) assay for the diagnosis of septic synovitis in horses. Can. J. Vet. Res..

[27] Oeser C., Pond M., Butcher P., Bedford Russell A., Henneke P., Laing K., Planche T., Heath P.T., Harris K. (2020). PCR for the detection of pathogens in neonatal early onset sepsis. PLoS One.

[28] Hackett E.S., Lunn D.P., Ferris R.A., Horohov D.W., Lappin M.R., McCue P.M. (2015). Detection of bacteraemia and host response in healthy neonatal foals. Equine Vet. J..

[29] Cheng A.P., Burnham P., Lee J.R., Cheng M.P., Suthanthiran M., Dadhania D., De Vlaminck I. (2019). A cell-free DNA metagenomic sequencing assay that integrates the host injury response to infection. Proc. Natl. Acad. Sci. USA.

[30] Pietrzak B., Kawacka I., Olejnik-Schmidt A., Schmidt M. (2023). Circulating Microbial Cell-Free DNA in Health and Disease. Int. J. Mol. Sci..

[31] Blauwkamp T.A., Thair S., Rosen M.J., Blair L., Lindner M.S., Vilfan I.D., Kawli T., Christians F.C., Venkatasubrahmanyam S., Wall G.D. (2019). Analytical and clinical validation of a microbial cell-free DNA sequencing test for infectious disease. Nat. Microbiol..

[32] Nielsen M.E., Søgaard K.K., Karst S.M., Krarup A.L., Nielsen H.L., Albertsen M. (2024). A new method using rapid Nanopore metagenomic cell-free DNA sequencing to diagnose bloodstream infections: a prospective observational study. medRxiv.

[33] Serpas L., Chan R.W.Y., Jiang P., Ni M., Sun K., Rashidfarrokhi A., Soni C., Sisirak V., Lee W.-S., Cheng S.H. (2019). *Dnase1l3* deletion causes aberrations in length and end-motif frequencies in plasma DNA. Proc. Natl. Acad. Sci. USA.

[34] Zhou Z., Ma M.-J.L., Chan R.W.Y., Lam W.K.J., Peng W., Gai W., Hu X., Ding S.C., Ji L., Zhou Q. (2023). Fragmentation landscape of cell-free DNA revealed by deconvolutional analysis of end motifs. Proc. Natl. Acad. Sci. USA.

[35] Thierry A.R. (2023). Circulating DNA fragmentomics and cancer screening. Cell Genom..

[36] Kowarsky M., Camunas-Soler J., Kertesz M., De Vlaminck I., Koh W., Pan W., Martin L., Neff N.F., Okamoto J., Wong R.J. (2017). Numerous uncharacterized and highly divergent microbes which colonize humans are revealed by circulating cell-free DNA. Proc. Natl. Acad. Sci. USA.

[37] Sanchez C., Roch B., Mazard T., Blache P., Dache Z.A.A., Pastor B., Pisareva E., Tanos R., Thierry A.R. (2021). Circulating nuclear DNA structural features, origins, and complete size profile revealed by fragmentomics. JCI Insight.

[38] Yu S.C.Y., Deng J., Qiao R., Cheng S.H., Peng W., Lau S.L., Choy L.Y.L., Leung T.Y., Wong J., Wong V.W.-S. (2023). Comparison of Single Molecule, Real-Time Sequencing and Nanopore Sequencing for Analysis of the Size, End-Motif, and Tissue-of-Origin of Long Cell-Free DNA in Plasma. Clin. Chem..

[39] Chang A., Mzava O., Lenz J.S., Cheng A.P., Burnham P., Motley S.T., Bennett C., Connelly J.T., Dadhania D.M., Suthanthiran M. (2021). Measurement Biases Distort Cell-Free DNA Fragmentation Profiles and Define the Sensitivity of Metagenomic Cell-Free DNA Sequencing Assays. Clin. Chem..

[40] Huang Y.-F., Chen Y.-J., Fan T.-C., Chang N.-C., Chen Y.-J., Midha M.K., Chen T.-H., Yang H.-H., Wang Y.-T., Yu A.L., Chiu K.P. (2018). Analysis of microbial sequences in plasma cell-free DNA for early-onset breast cancer patients and healthy females. BMC Med. Genomics.

[41] Chen H., Zheng Y., Zhang X., Liu S., Yin Y., Guo Y., Wang X., Zhang Y., Zhao C., Gai W., Wang H. (2024). Clinical evaluation of cell-free and cellular metagenomic next-generation sequencing of infected body fluids. J. Adv. Res..

[42] Wang G., Lam W.K.J., Ling L., Ma M.-J.L., Ramakrishnan S., Chan D.C.T., Lee W.-S., Cheng S.H., Chan R.W.Y., Yu S.C.Y. (2023). Fragment Ends of Circulating Microbial DNA as Signatures for Pathogen Detection in Sepsis. Clin. Chem..

[43] Jiang P., Sun K., Peng W., Cheng S.H., Ni M., Yeung P.C., Heung M.M.S., Xie T., Shang H., Zhou Z. (2020). Plasma DNA End-Motif Profiling as a Fragmentomic Marker in Cancer, Pregnancy, and Transplantation. Cancer Discov..

[44] Colmer S.F., Luethy D., Abraham M., Stefanovski D., Hurcombe S.D. (2021). Utility of cell-free DNA concentrations and illness severity scores to predict survival in critically ill neonatal foals. PLoS One.

[45] Hobbs K.J., Cooper B.L., Dembek K., Sheats M.K. (2024). Investigation of Extracted Plasma Cell-Free DNA as a Biomarker in Foals with Sepsis. Vet. Sci..

[46] Burnham P., Kim M.S., Agbor-Enoh S., Luikart H., Valantine H.A., Khush K.K., De Vlaminck I. (2016). Single-stranded DNA library preparation uncovers the origin and diversity of ultrashort cell-free DNA in plasma. Sci. Rep..

[47] Gihawi A., Ge Y., Lu J., Puiu D., Xu A., Cooper C.S., Brewer D.S., Pertea M., Salzberg S.L. (2023). Major data analysis errors invalidate cancer microbiome findings. mBio.

[48] Wood D.E., Lu J., Langmead B. (2019). Improved metagenomic analysis with Kraken 2. Genome Biol..

[49] Davis N.M., Proctor D.M., Holmes S.P., Relman D.A., Callahan B.J. (2018). Simple statistical identification and removal of contaminant sequences in marker-gene and metagenomics data. Microbiome.

[50] Lu J., Breitwieser F.P., Thielen P., Salzberg S.L. (2017). Bracken: estimating species abundance in metagenomics data. PeerJ Comput. Sci..

[51] Theelen M.J.P., Wilson W.D., Byrne B.A., Edman J.M., Kass P.H., Mughini-Gras L., Magdesian K.G. (2020). Differences in isolation rate and antimicrobial susceptibility of bacteria isolated from foals with sepsis at admission and after ≥48 hours of hospitalization. J. Vet. Intern. Med..

[52] Jin X., Wang Y., Xu J., Li Y., Cheng F., Luo Y., Zhou H., Lin S., Xiao F., Zhang L. (2023). Plasma cell-free DNA promise monitoring and tissue injury assessment of COVID-19. Mol. Genet. Genomics..

[53] Jing Q., Leung C.H.C., Wu A.R. (2022). Cell-Free DNA as Biomarker for Sepsis by Integration of Microbial and Host Information. Clin. Chem..

[54] Charoensappakit A., Sae-Khow K., Rattanaliam P., Vutthikraivit N., Pecheenbuvan M., Udomkarnjananun S., Leelahavanichkul A. (2023). Cell-free DNA as diagnostic and prognostic biomarkers for adult sepsis: a systematic review and meta-analysis. Sci. Rep..

[55] Kung C.-T., Hsiao S.-Y., Tsai T.-C., Su C.-M., Chang W.-N., Huang C.-R., Wang H.-C., Lin W.-C., Chang H.-W., Lin Y.-J. (2012). Plasma nuclear and mitochondrial DNA levels as predictors of outcome in severe sepsis patients in the emergency room. J. Transl. Med..

[56] Yan H.P., Li M., Lu X.L., Zhu Y.M., Ou-Yang W.-X., Xiao Z.H., Qiu J., Li S.J. (2018). Use of plasma mitochondrial DNA levels for determining disease severity and prognosis in pediatric sepsis: a case control study. BMC Pediatr..

[57] Henry B.M., de Oliveira M.H.S., Cheruiyot I., Benoit J., Rose J., Favaloro E.J., Lippi G., Benoit S., Pode Shakked N. (2022). Cell-Free DNA, Neutrophil extracellular traps (NETs), and Endothelial Injury in Coronavirus Disease 2019- (COVID-19-) Associated Acute Kidney Injury. Mediators Inflamm..

[58] Corley K.T.T., Pearce G., Magdesian K.G., Wilson W.D. (2007). Bacteraemia in neonatal foals: clinicopathological differences between Gram-positive and Gram-negative infections, and single organism and mixed infections. Equine Vet. J..

[59] Grumaz C., Hoffmann A., Vainshtein Y., Kopp M., Grumaz S., Stevens P., Decker S.O., Weigand M.A., Hofer S., Brenner T., Sohn K. (2020). Rapid Next-Generation Sequencing-Based Diagnostics of Bacteremia in Septic Patients. J. Mol. Diagn..

[60] Grumaz S., Stevens P., Grumaz C., Decker S.O., Weigand M.A., Hofer S., Brenner T., von Haeseler A., Sohn K. (2016). Next-generation sequencing diagnostics of bacteremia in septic patients. Genome Med..

[61] Park S.Y., Chang E.J., Ledeboer N., Messacar K., Lindner M.S., Venkatasubrahmanyam S., Wilber J.C., Vaughn M.L., Bercovici S., Perkins B.A., Nolte F.S. (2023). Plasma Microbial Cell-Free DNA Sequencing from over 15,000 Patients Identified a Broad Spectrum of Pathogens. J. Clin. Microbiol..

[62] Grumaz S., Grumaz C., Vainshtein Y., Stevens P., Glanz K., Decker S.O., Hofer S., Weigand M.A., Brenner T., Sohn K. (2019). Enhanced Performance of Next-Generation Sequencing Diagnostics Compared With Standard of Care Microbiological Diagnostics in Patients Suffering From Septic Shock. Crit. Care Med..

[63] Hartwig C., Drechsler S., Vainshtein Y., Maneth M., Schmitt T., Ehling-Schulz M., Osuchowski M., Sohn K. (2023). From Gut to Blood: Spatial and Temporal Pathobiome Dynamics during Acute Abdominal Murine Sepsis. Microorganisms.

[64] Yan H.P., Li M., Lu X.L., Zhu Y.M., Ou-Yang W.-X., Xiao Z.H., Qiu J., Li S.J. (2018). Use of plasma mitochondrial DNA levels for determining disease severity and prognosis in pediatric sepsis: a case control study. BMC Pediatr..

[65] Di Caro V., Walko T.D., Bola R.A., Hong J.D., Pang D., Hsue V., Au A.K., Halstead E.S., Carcillo J.A., Clark R.S.B., Aneja R.K. (2016). Plasma Mitochondrial DNA--a Novel DAMP in Pediatric Sepsis. Shock.

[66] Diaz L.A., Bardelli A. (2014). Liquid Biopsies: Genotyping Circulating Tumor DNA. J. Clin. Oncol..

[67] Kalantar K.L., Neyton L., Abdelghany M., Mick E., Jauregui A., Caldera S., Serpa P.H., Ghale R., Albright J., Sarma A. (2022). Integrated host-microbe plasma metagenomics for sepsis diagnosis in a prospective cohort of critically ill adults. Nat. Microbiol..

[68] Serpa P.H., Deng X., Abdelghany M., Crawford E., Malcolm K., Caldera S., Fung M., McGeever A., Kalantar K.L., Lyden A. (2022). Metagenomic prediction of antimicrobial resistance in critically ill patients with lower respiratory tract infections. Genome Med..

[69] Cohen J.F., Korevaar D.A., Altman D.G., Bruns D.E., Gatsonis C.A., Hooft L., Irwig L., Levine D., Reitsma J.B., de Vet H.C.W., Bossuyt P.M.M. (2016). STARD 2015 guidelines for reporting diagnostic accuracy studies: explanation and elaboration. BMJ Open.

[70] Sounderajah V., Guni A., Liu X., Collins G.S., Karthikesalingam A., Markar S.R., Golub R.M., Denniston A.K., Shetty S., Moher D. (2025). The STARD-AI reporting guideline for diagnostic accuracy studies using artificial intelligence. Nat. Med..

[71] Troll C.J., Kapp J., Rao V., Harkins K.M., Cole C., Naughton C., Morgan J.M., Shapiro B., Green R.E. (2019). A ligation-based single-stranded library preparation method to analyze cell-free DNA and synthetic oligos. BMC Genom..

[72] (2014). BBMap: A Fast, Accurate, Splice-Aware Aligner. https://escholarship.org/uc/item/1h3515gn.

[73] Dai H., Guan Y. (2020). Nubeam-dedup: a fast and RAM-efficient tool to de-duplicate sequencing reads without mapping. Bioinformatics.

[74] Chen S., Zhou Y., Chen Y., Gu J. (2018). fastp: an ultra-fast all-in-one FASTQ preprocessor. Bioinformatics.

[75] Schubert M., Lindgreen S., Orlando L. (2016). AdapterRemoval v2: rapid adapter trimming, identification, and read merging. BMC Res. Notes.

[76] Langmead B., Salzberg S.L. (2012). Fast gapped-read alignment with Bowtie 2. Nat. Methods.

[77] Wright R.J., Comeau A.M., Langille M.G.I. (2023). From defaults to databases: parameter and database choice dramatically impact the performance of metagenomic taxonomic classification tools. Microb. Genom..

[78] Lu J., Rincon N., Wood D.E., Breitwieser F.P., Pockrandt C., Langmead B., Salzberg S.L., Steinegger M. (2022). Metagenome analysis using the Kraken software suite. Nat. Protoc..

[79] Shannon C.E. (1948). A mathematical theory of communication. Bell Syst. Tech. J..

