# Supplementary material for: Bacterial cell-free DNA profiling reveals the co-elevation of multiple bacteria in newborn foals with suspected sepsis
Source: iScience. 2025 Nov 11;28(12):114005. doi: 10.1016/j.isci.2025.114005 (PMC12753253; doi:10.1016/j.isci.2025.114005)
Supplement: Document S1. Figures S1–S13, Tables S1, S2, S8, and Table S11 [file mmc1.pdf]

iScience, Volume *28*

Supplemental information

Bacterial cell-free DNA profiling reveals the co-elevation of multiple bacteria in newborn foals with suspected sepsis

Li-Ting Chen, Emmy Wesdorp, Myrthe Jager, Esther W. Siegers, Mathijs J.P. Theelen, Nicolle Besselink, Carlo Vermeulen, Aldert L. Zomer, Els M. Broens, Jaap A. Wagenaar, and Jeroen de Ridder

Supplementary Figures S1-S13

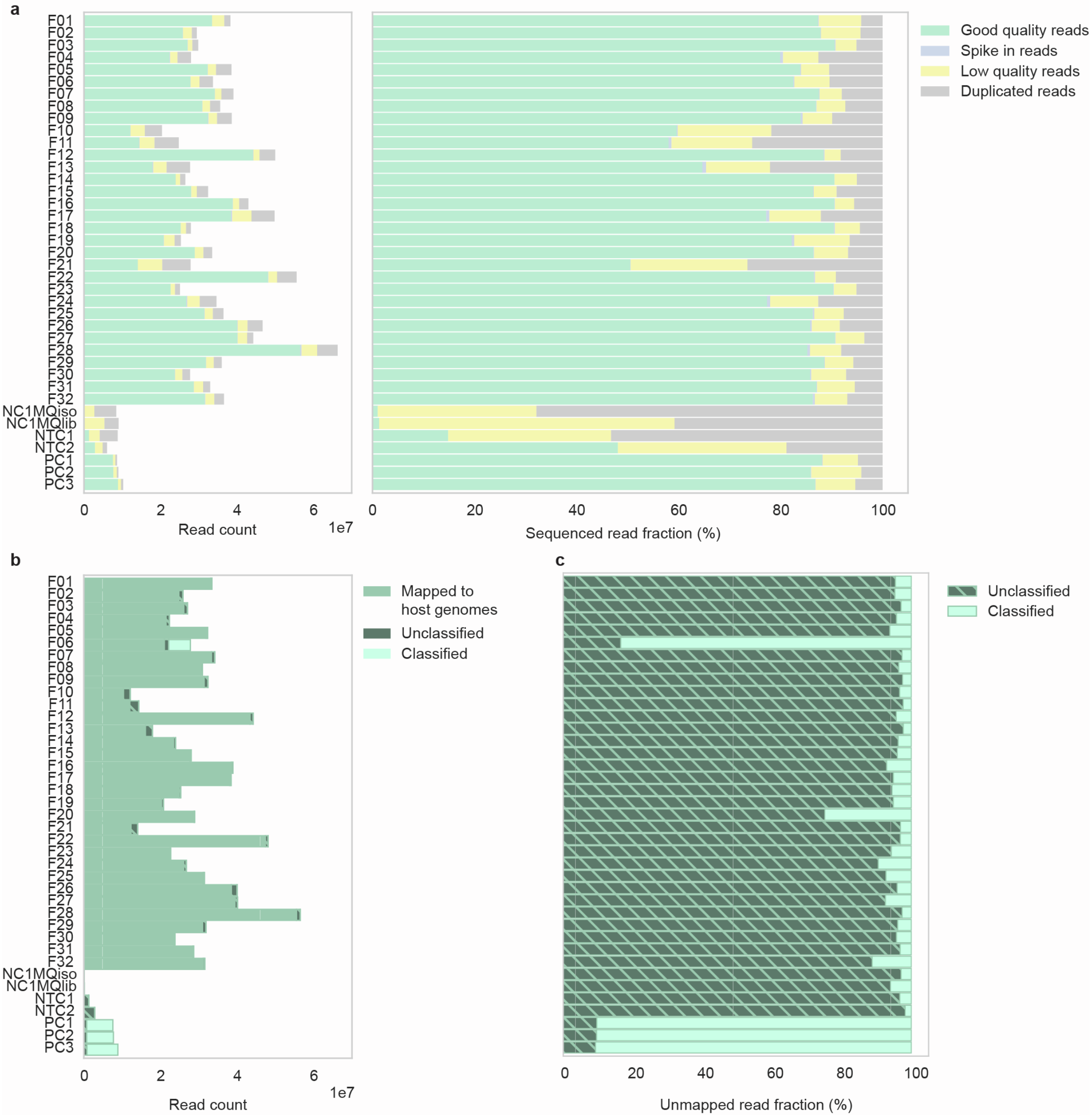

Supplementary Figure S1. Overview of sequencing read count, read-quality composition, host mapping rate and metagenomics classification rate of foal cfDNA and control samples. Related to Figure 1b.

a. Number (left) and proportion (right) of paired-end Illumina sequenced reads in each biological and control samples represented in stacked bar plots. Colors represent the fractions of spike-in reads, duplicated reads, low-quality reads, and high-quality reads.

b. Number of read passed filtering in each sample represented in stacked bar plots. Colors indicate the fractions of reads mapped to the host genome, taxonomically classified and unclassified reads.

c. The relative proportion of reads not mapped to host in each sample represented in stacked bar plots. Colors indicate the fractions of taxonomically classified and unclassified reads.

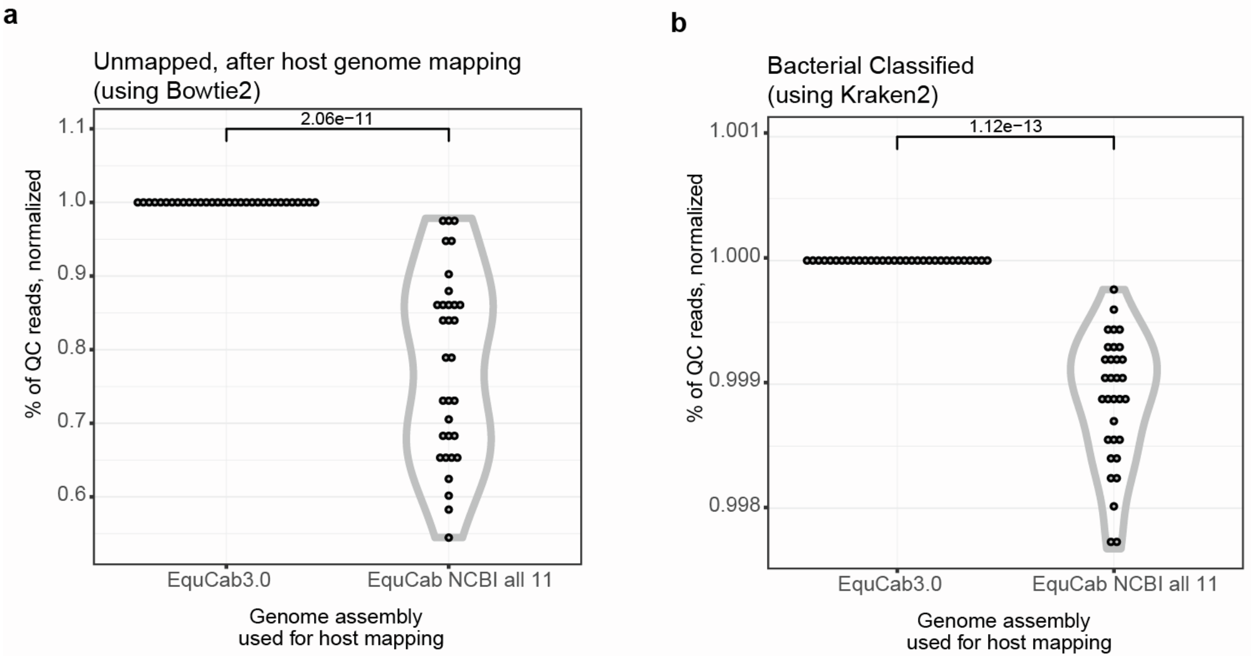

Supplementary Figure S2. Comparison of read host mapping and bacterial classification using different horse genome reference genomes. Related to Figure 1b.

a. Percentage of quality-controlled reads remaining after mapping to the human reference genome using the cfFBI workflow. Two conditions were compared: using the EquCab3 reference genome alone, and using a compendium that includes all available horse genomes on NCBI (a total of 11 genomes; EquCab NCBI all 11). The results are normalized to those obtained with EquCab3. Statistical analysis was conducted using one-tailed paired t-tests after normalization.

b. Percentage of quality-controlled reads classified as bacterial by Kraken2, after subtracting host reads via reference genome mapping. Two conditions were compared: using the EquCab3 reference genome alone, and using a compendium that includes all available horse genomes on NCBI (a total of 11 genomes; EquCab NCBI all 11). The Kraken2 bacterial classification results are normalized to those obtained with the EquCab3 reference genome. Statistical analysis was conducted using one-tailed paired t-tests after normalization.

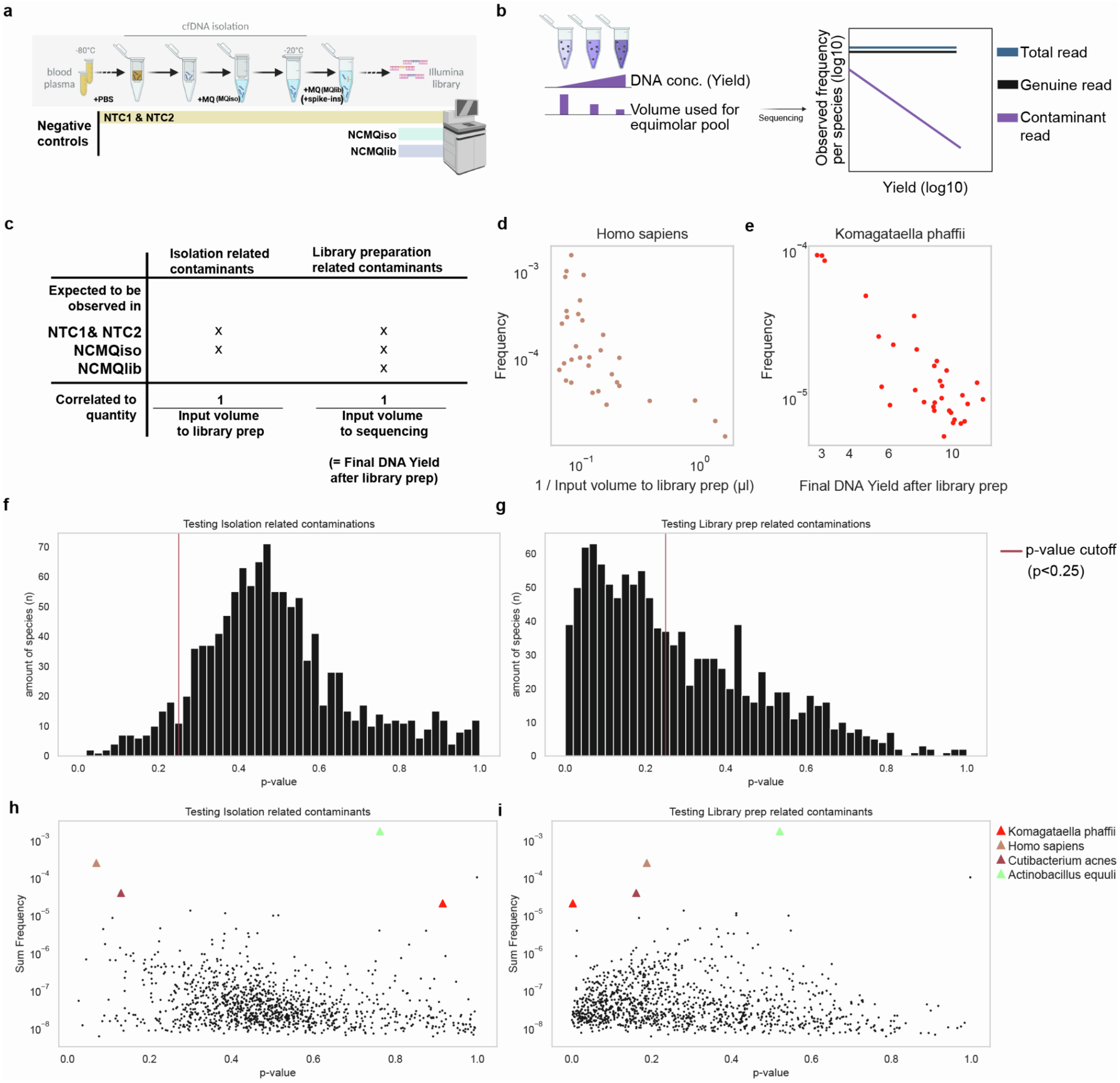

Supplementary Figure S3. Schematic of negative controls and species-level contaminant identification showing inverse frequency–yield relationships across cfDNA isolation and library-preparation steps. Related to Figure 2.

a. Schematic of four negative controls used in our study: Non-Template Controls (NTC1, NTC2), which underwent cfDNA DNA isolation and NGS library preparation, and Nuclease-Free water controls (NC1MQiso, NC1MQlib), which underwent only NGS library preparation.

b. Contaminants in our samples may have been introduced during cfDNA isolation or sequencing library preparation. Contaminant DNA is expected to be present in low, relatively uniform concentrations across laboratory equipment and kits, leading to similar levels across samples. In contrast, cfDNA concentration - and thus the yield from cfDNA isolation or library preparation - can vary significantly between samples. Consequently, the expected frequency of contaminant DNA decreases as the total DNA (yield) in the sample increases (purple), while the frequency of non-contaminant DNA remains consistent (black). Figure adapted from (Davis et al. 2018) [S1].

c. Table presents the expected cfDNA isolation and library preparation contaminants in each negative control sample (top), along with how input volumes serve as an inverse proxy for yield (bottom).

d. Dotplot showing the relative frequency of *Homo sapiens* reads, an identified cfDNA isolation-related contaminant, versus the input volume used for library preparation (inverse proxy for cfDNA yield) across 32 samples.

e. Dotplot showing the relative frequency of *Komagataella phaffi* reads, a proven library preparation-related contaminant, versus the input volume used for pooling (inverse proxy for library preparation yield) across 32 samples.

f. Histogram of p-values derived from the decontam method for cfDNA isolation-related contaminants in all species tested (n = 1167). The p-value detection classification threshold is set at 0.25, indicated by the red vertical line; species falling below this threshold are identified as cfDNA isolation-related contaminants.

g. Histogram of p-values derived from the decontam method for library preparation-related contaminants in all species tested (n = 1167). The p-value detection classification threshold is set at 0.25, indicated by the red vertical line; species falling below this threshold are identified as library preparation-related contaminants.

h. Scatter plot displaying the summed frequency across all foals against p-values derived from the decontam method for cfDNA isolation-related contaminants. Contaminants *Homo sapiens* ($p < 0.25$) and *Cutibacterium acnes* ($p < 0.25$) are highlighted, indicated by red and brown triangles, respectively. The NGS library preparation-associated contaminant *Komagataella phaffi* (not significant) is also marked with a triangle. Additionally, *Actinobacillus equuli*, a highly abundant microbial species (frequently observed pathogen in previous research) that is not significantly detected as a contaminant, is represented by a green triangle.

i. Scatter plot displaying the summed frequency across all foals against p-values derived from the decontam method for library preparation-related contaminants, highlighting contaminants. Contaminants *Homo sapiens* ($p < 0.25$) and *Cutibacterium acnes* ($p < 0.25$) are highlighted, indicated by red and brown triangles, respectively. The library preparation-associated contaminant *Komagataella phaffi* ($p < 0.25$) is also marked with a triangle. Additionally, *Actinobacillus equuli*, a highly abundant pathogen that is not significant, is represented by a green triangle.

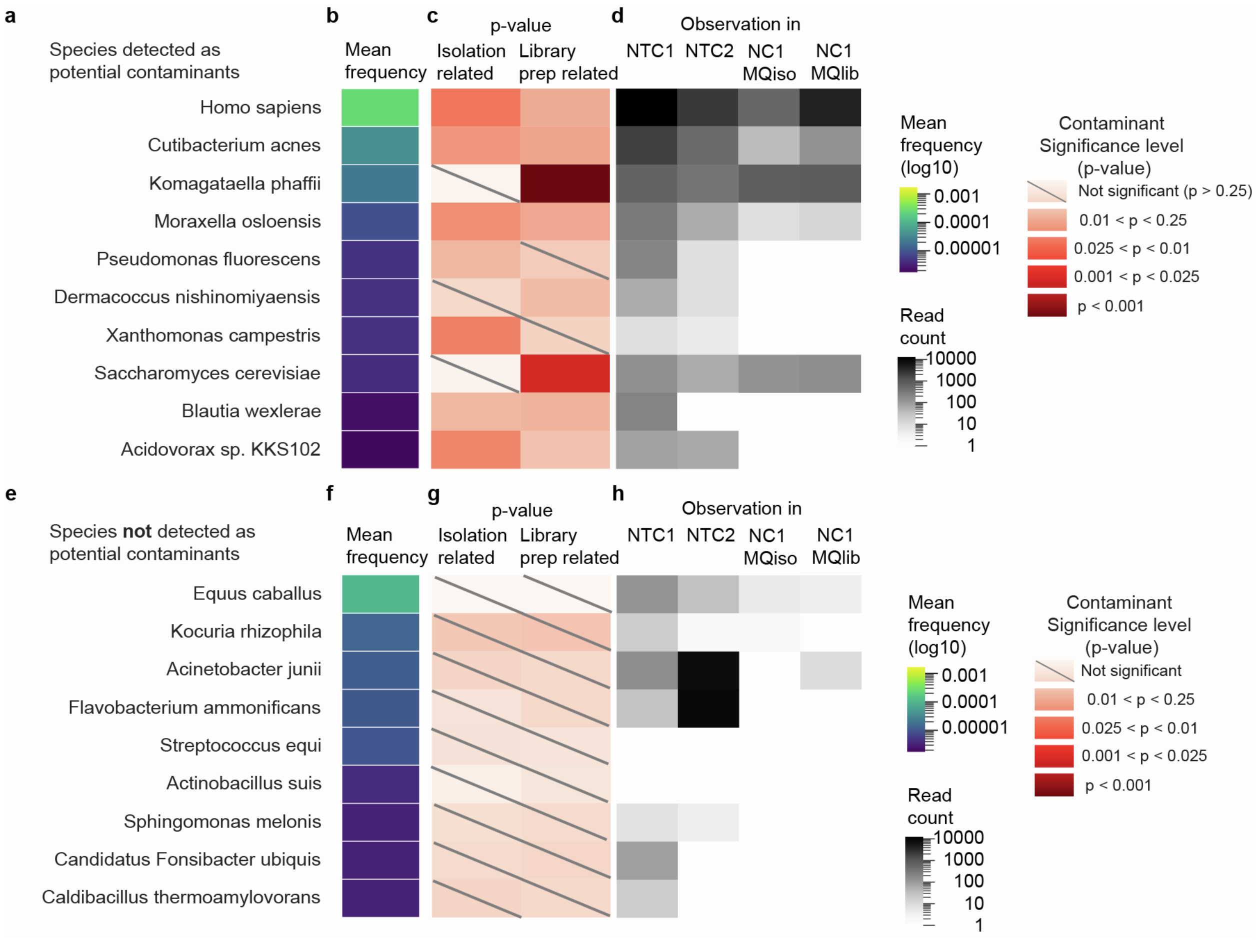

Supplementary Figure S4. Top ten species detected as contaminants and top ten species not detected as contaminants with their presence in negative controls. Related to Figure 2.

a. Top 10 species detected as potential contaminants sorted by mean relative abundance.

b. Heatmap illustrates the mean frequency of potential contaminant species listed in **a.** The color reflects the log10 mean frequency of each species across all foal samples.

c. Heatmap displays p-values indicating whether the contaminants are associated with the cfDNA isolation process or the library preparation process. Significance levels range from not significant (light pink, p > 0.25) to highly significant (dark red).

d. Observations of contaminant species in various negative control conditions: NTC1, NTC2, NC1MQiso, and NC1MQlib. The color intensity reflects the log10 count frequency.

e. Top 10 species non-contaminant species sorted by mean relative abundance.

f. Heatmap illustrates the mean frequency of potential non-contaminant species listed in **e.** The color reflects the log10 mean frequency of each species across all foal samples.

g. Heatmap of p-values indicating whether the species not detected as contaminants are related to the isolation process or the library preparation process. All were p> 0.25.

h. Observations of non-contaminant species in various negative control conditions: NTC1, NTC2, NC1MQiso, and NC1MQlib. The color intensity reflects the log10 count frequency.

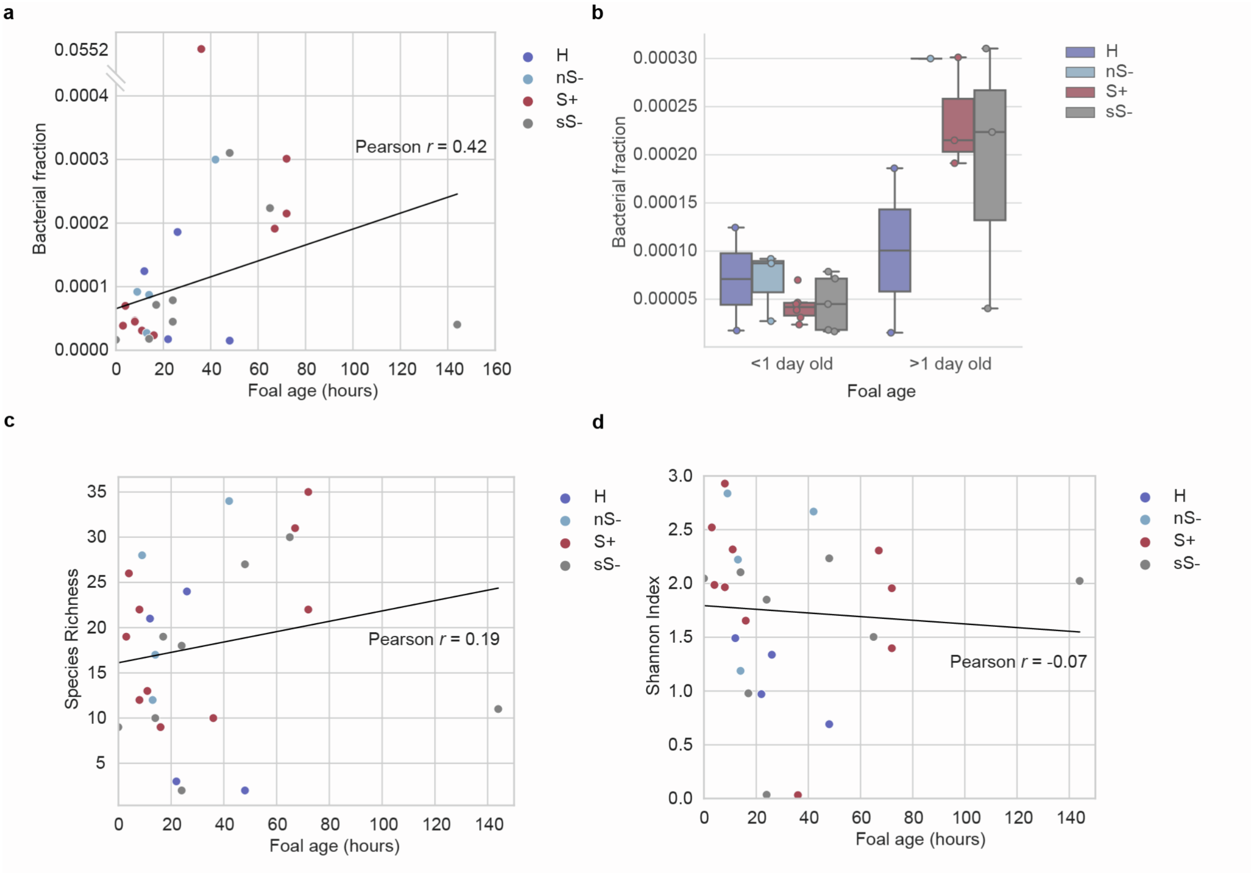

Supplementary Figure S5. Relationships between foal age, disease status, and bacterial load/diversity metrics. Related to Figure 2.

a. Scatter plot showing the bacterial fraction in relation to foal age at presentation (hours). Data points are color-coded based on disease status. A weak Pearson correlation (*r*=0.42) was calculated excluding outlier data point at bacterial fraction = 0.0552 to avoid strong influence on fitting caused by the outlier. If including this data point, the Pearson correlation is *r*=0.03.

b. Box plot illustrates the bacterial fraction in foals less than one day old compared to those more than one day old. Data points are grouped by health status as shown in **a.**, with whiskers extending to the rest of the distribution within 1.5 times the interquartile range.

c. Scatter plot depicting species richness in relation to foal age at presentation (hours). Data points are color-coded by health status as in **a.**. Weak correlation was observed (Pearson correlation *r*=0.19).

d. Scatter plot showing the Shannon index in relation to foal age at presentation (hours). Data points are color-coded by health status as in **a.**. Weak negative correlation was observed (Pearson correlation *r*=0.19).

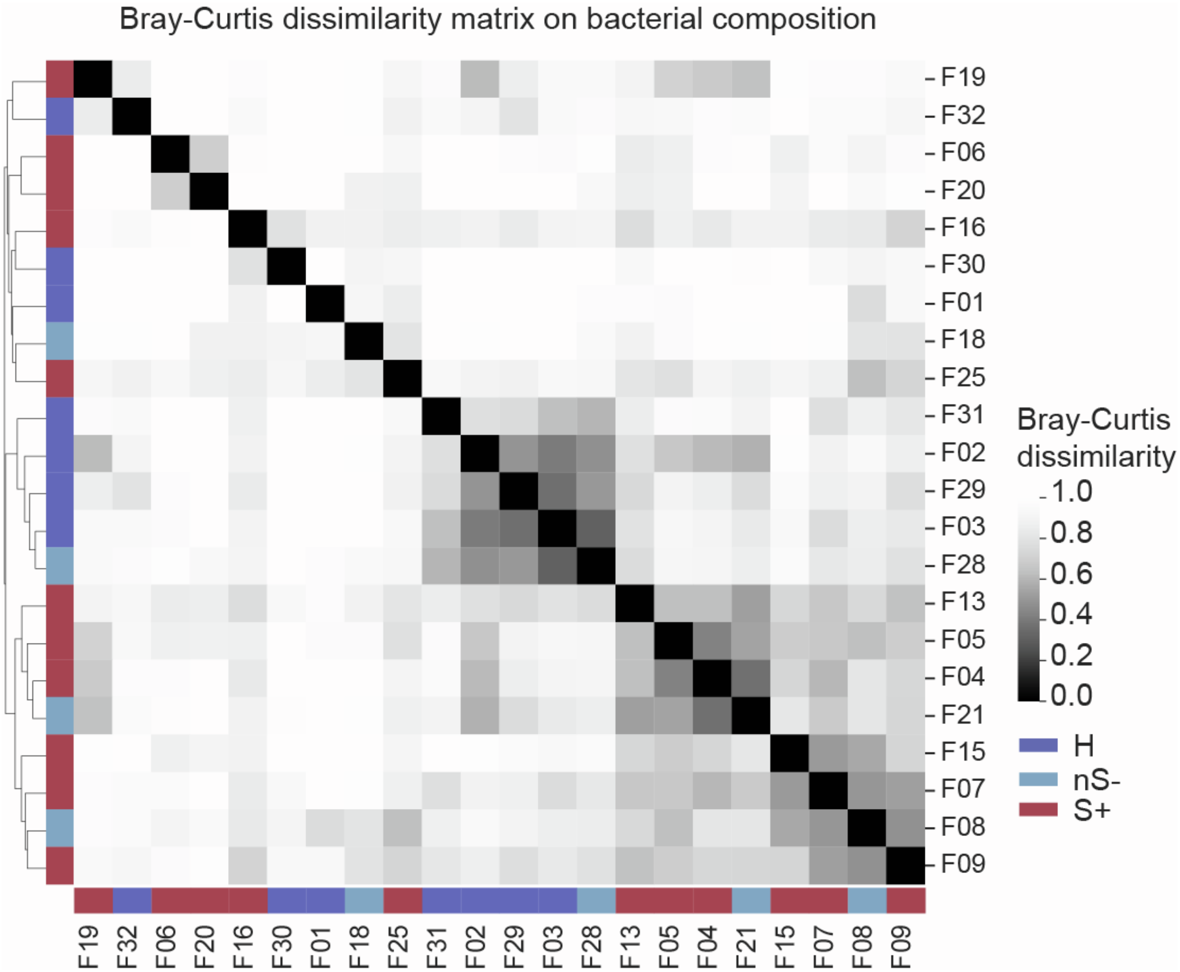

Supplementary Figure S6. Bray-Curtis dissimilarity of bacterial composition between samples with disease status annotation. Related to Figure 2.

Heat map of pairwise Bray–Curtis dissimilarity (0 = identical; 1 = maximally different) between foal samples computed from species-level relative abundances. Rows and columns are hierarchically clustered (average linkage), with the dendrogram on the left. Colored side and bottom bars denote clinical categories: H, nS−, and S+. Darker tiles indicate greater between-sample dissimilarity.

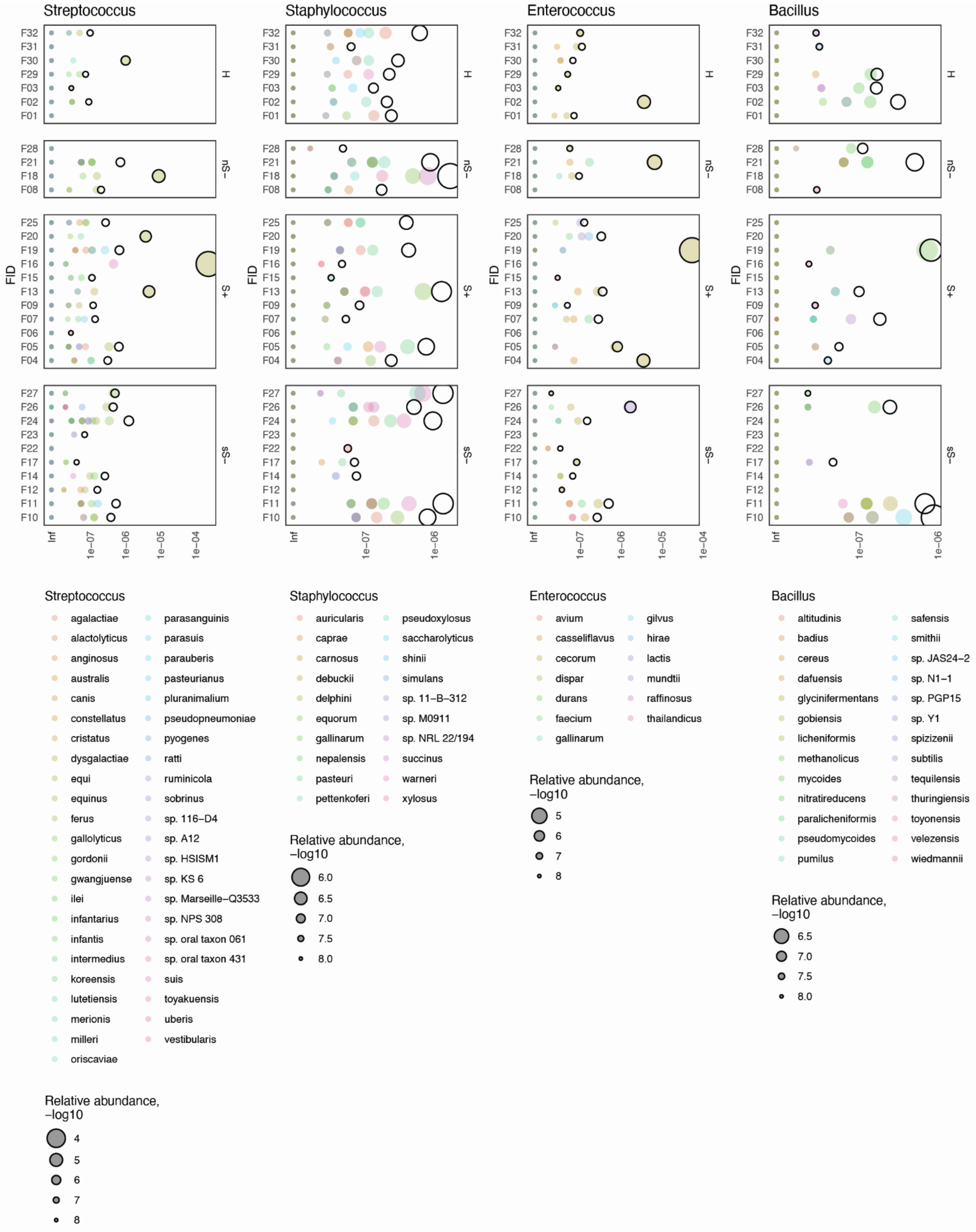

Supplementary Figure S7. Relative abundance of pathogenic Gram-positive bacterial species and genera. Related to Figure 3.

Relative abundance (x-axis) of species from four selected Gram-positive pathogenic bacterial genera. Total relative abundance of these genera (sum of species) across samples is represented by black hollow circles. Each dot color represents a different species, while the dot size indicates their relative abundance. For species with no observations, relative abundance is indicated as infinity (Inf).

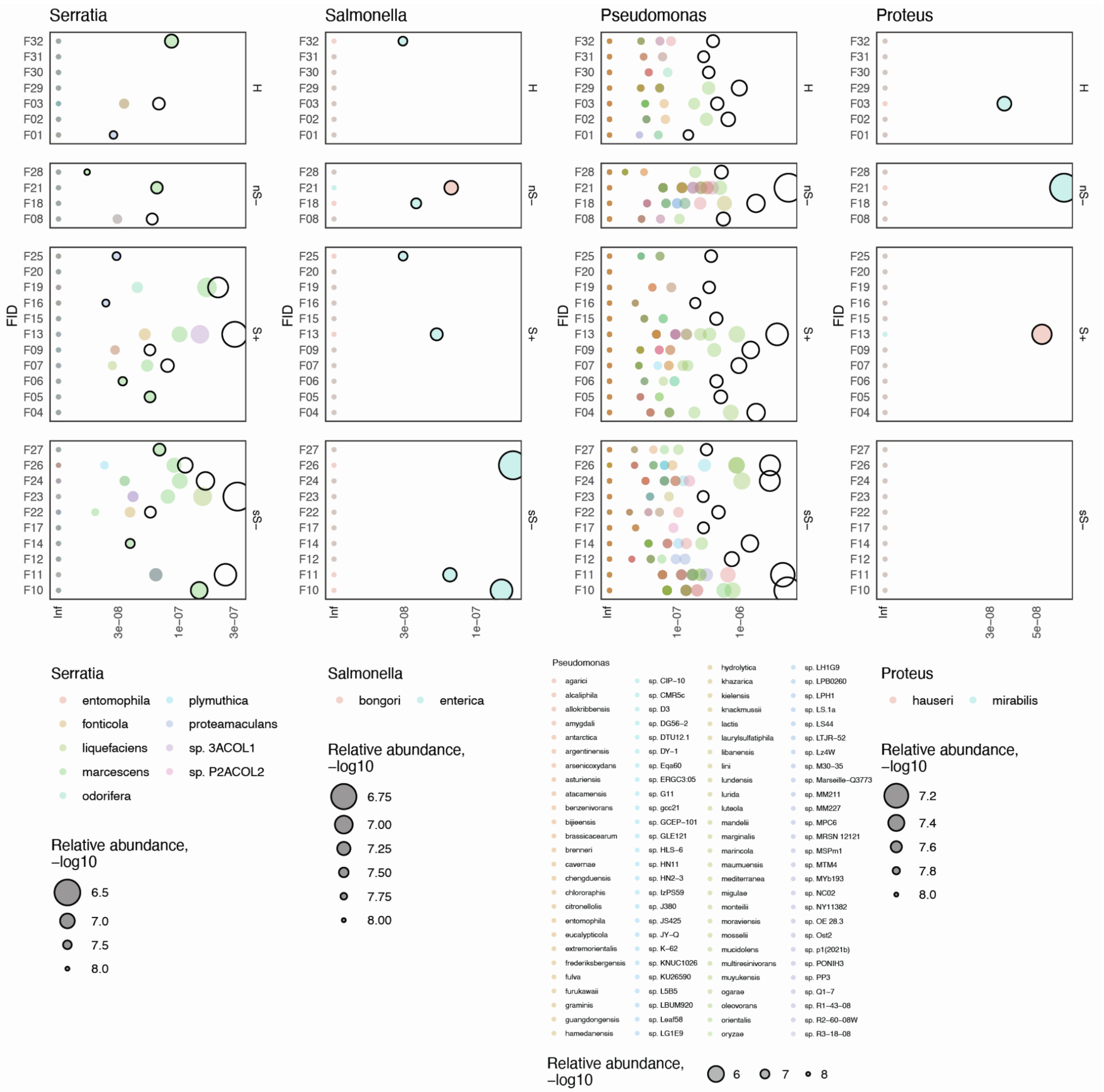

Supplementary Figure S8. Relative abundance of pathogenic Gram-negative bacterial species and genera. Related to Figure 3.

Relative abundance (x-axis) of species from twelve selected Gram-negative pathogenic bacterial genera. Total relative abundance of these genera (sum of species) across samples is represented by black hollow circles. Each dot color represents a different species, while the dot size indicates their relative abundance. For species with no observations, relative abundance is indicated as infinity (Inf).

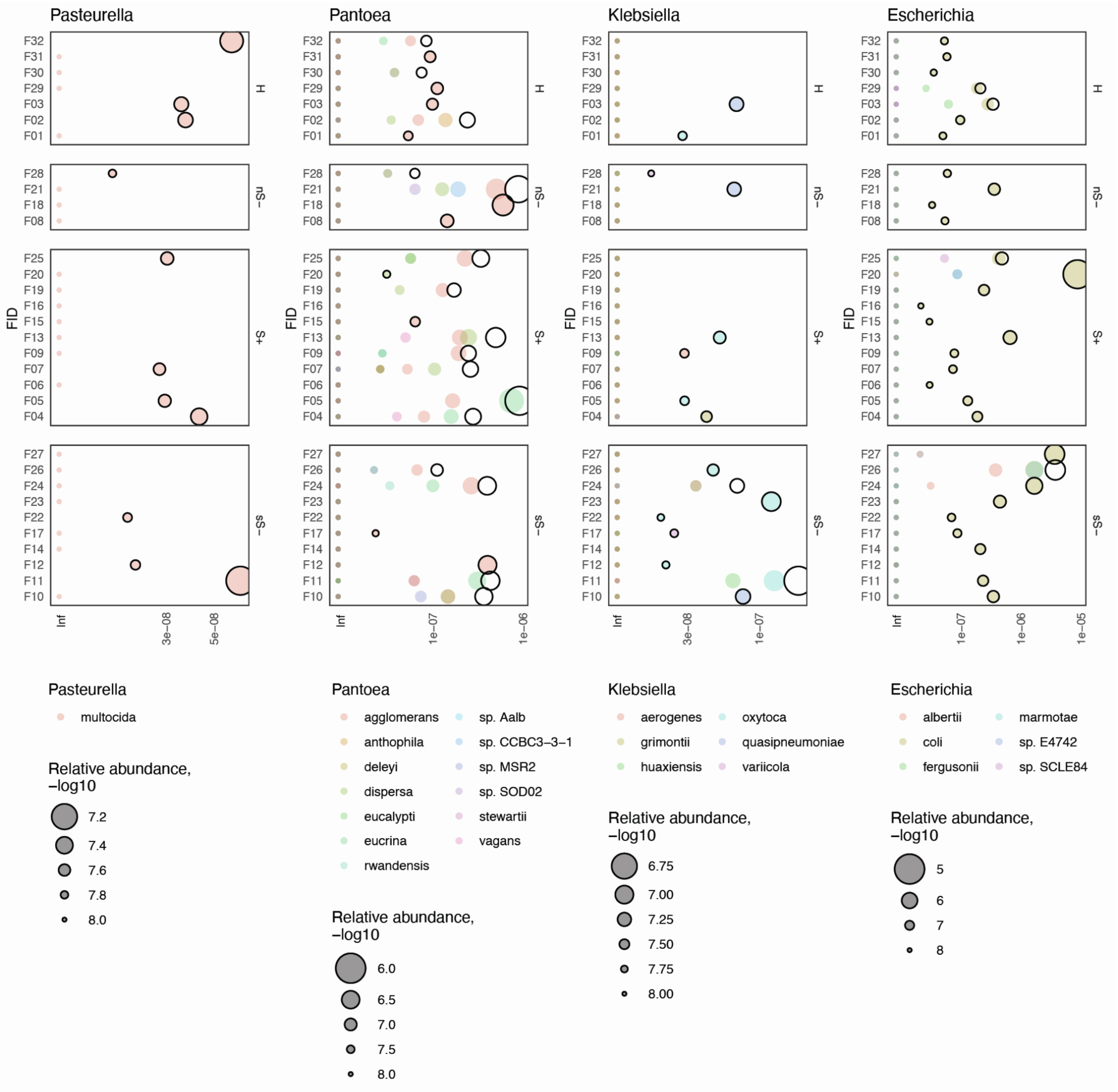

Supplementary Figure S9. Relative abundance of pathogenic Gram-negative bacterial species and genera. Related to Figure 3.

Relative abundance (x-axis) of species from twelve selected Gram-negative pathogenic bacterial genera. Total relative abundance of these genera (sum of species) across samples is represented by black hollow circles. Each dot color represents a different species, while the dot size indicates their relative abundance. For species with no observations, relative abundance is indicated as infinity (Inf).

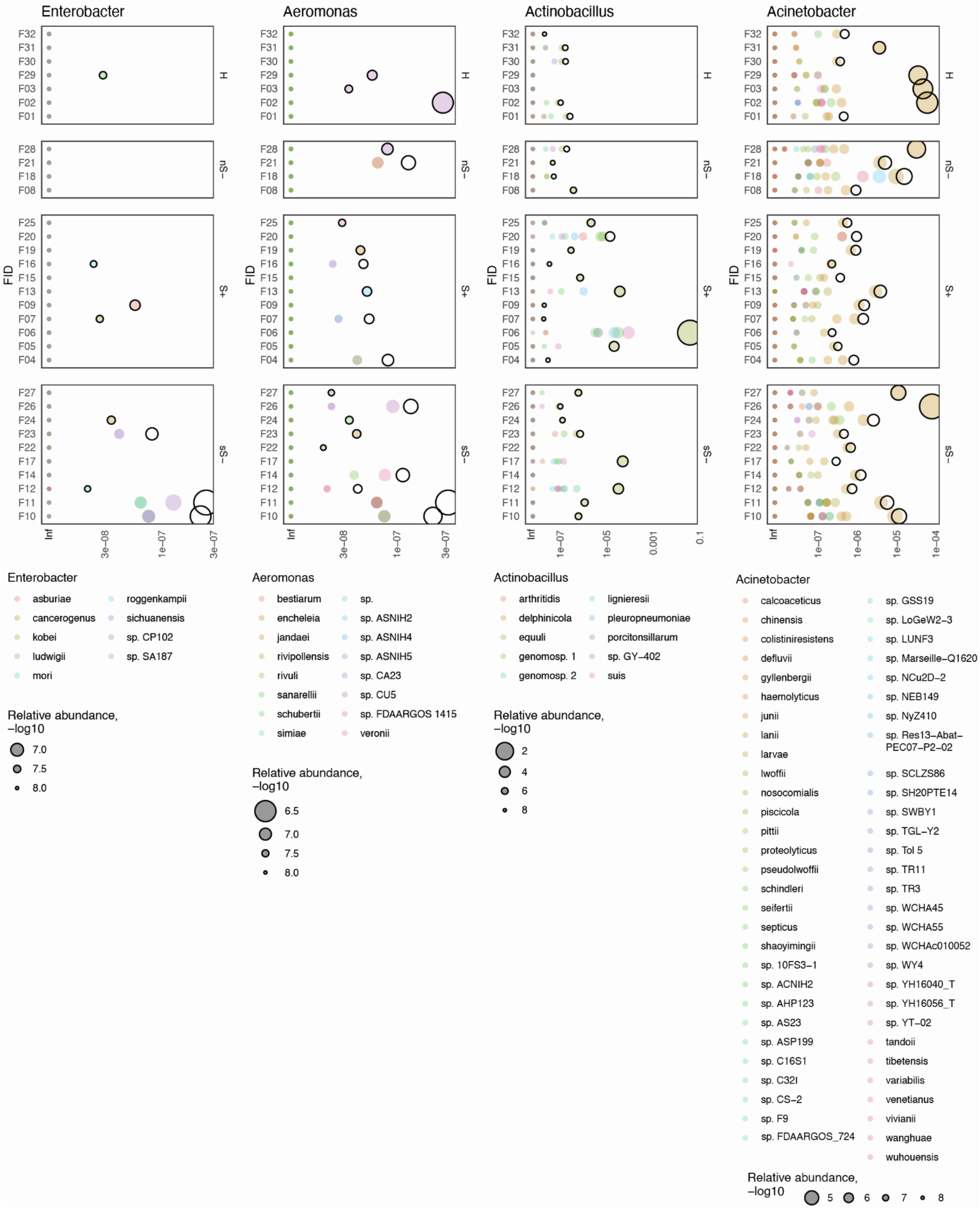

Supplementary Figure S10. Relative abundance of pathogenic Gram-negative bacterial species and genera. Related to Figure 3.

Relative abundance (x-axis) of species from twelve selected Gram-negative pathogenic bacterial genera. Total relative abundance of these genera (sum of species) across samples is represented by black hollow circles. Each dot color represents a different species, while the dot size indicates their relative abundance. For species with no observations, relative abundance is indicated as infinity (Inf).

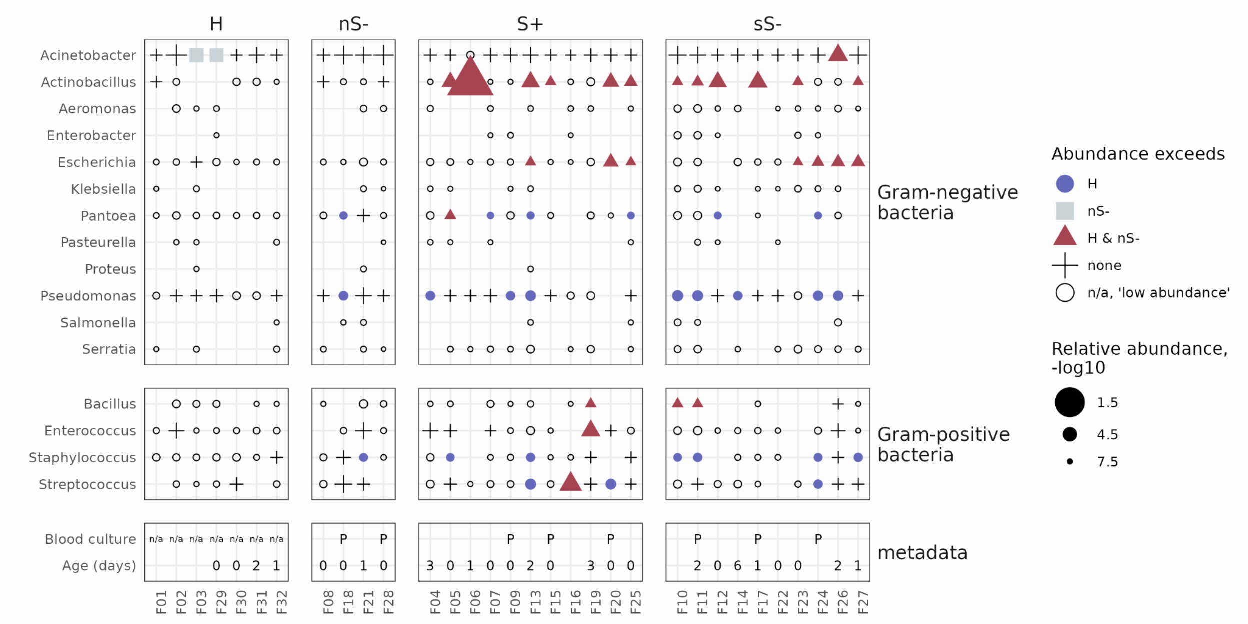

Supplementary Figure S11. Relative abundance of pathogenic bacterial species and genera compared to H and(or) nS- groups. Related to Figure 3.

Dotplot displaying the detection of the 16 most frequently cultured pathogenic genera. Gram-negative species are shown at the top, and Gram-positive species are shown in the middle. Each dot on the plot represents a genus that was detected with at least 1 read. The different symbols and colors convey the relative abundance of the genera in comparison to healthy (H) foals and nS- foals: Blue circles indicate genera with a relative abundance higher than in H foals. Light-gray squares indicate genera with a relative abundance higher than in nS- foals. Red triangles denote genera with a relative abundance higher than in H and nS- foals. White circles represent genera detected at low abundance (fewer than 10 reads) and therefore not compared to either H or nS- foals. Plus signs ('+') represent genera that are not elevated compared to either healthy (H) or non-suppurative (nS-) foals. Metadata is displayed at the bottom, including blood culture results ('P' for positive, 'n/a' for not performed) and age at hospital presentation, if known.

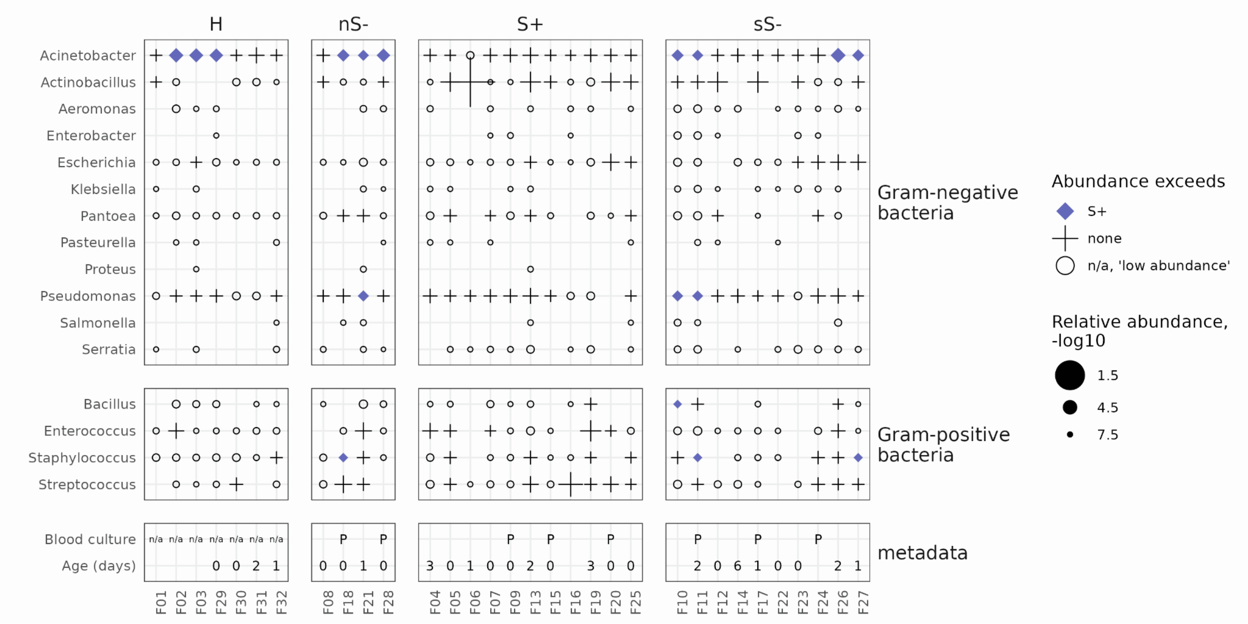

Supplementary Figure S12. Relative abundance of pathogenic bacterial species and genera compared to S+. Related to Figure 3.

Dotplot displaying the detection of the 16 most frequently cultured pathogenic genera. Gram-negative species are shown at the top, and Gram-positive species are shown in the middle. Each dot on the plot represents a genus that was detected with at least 1 read. The different symbols and colors convey the relative abundance of the genera in comparison to healthy (H) foals and nS- foals: Blue diamonds indicate genera with a relative abundance higher than in S+ foals. White circles represent genera detected at low abundance (fewer than 10 reads) and therefore not compared to either S+ foals. Plus signs ('+') represent genera that are not elevated compared to S+ foals. Metadata is displayed at the bottom, including blood culture results ('P' for positive, 'n/a' for not performed) and age at hospital presentation, if known.

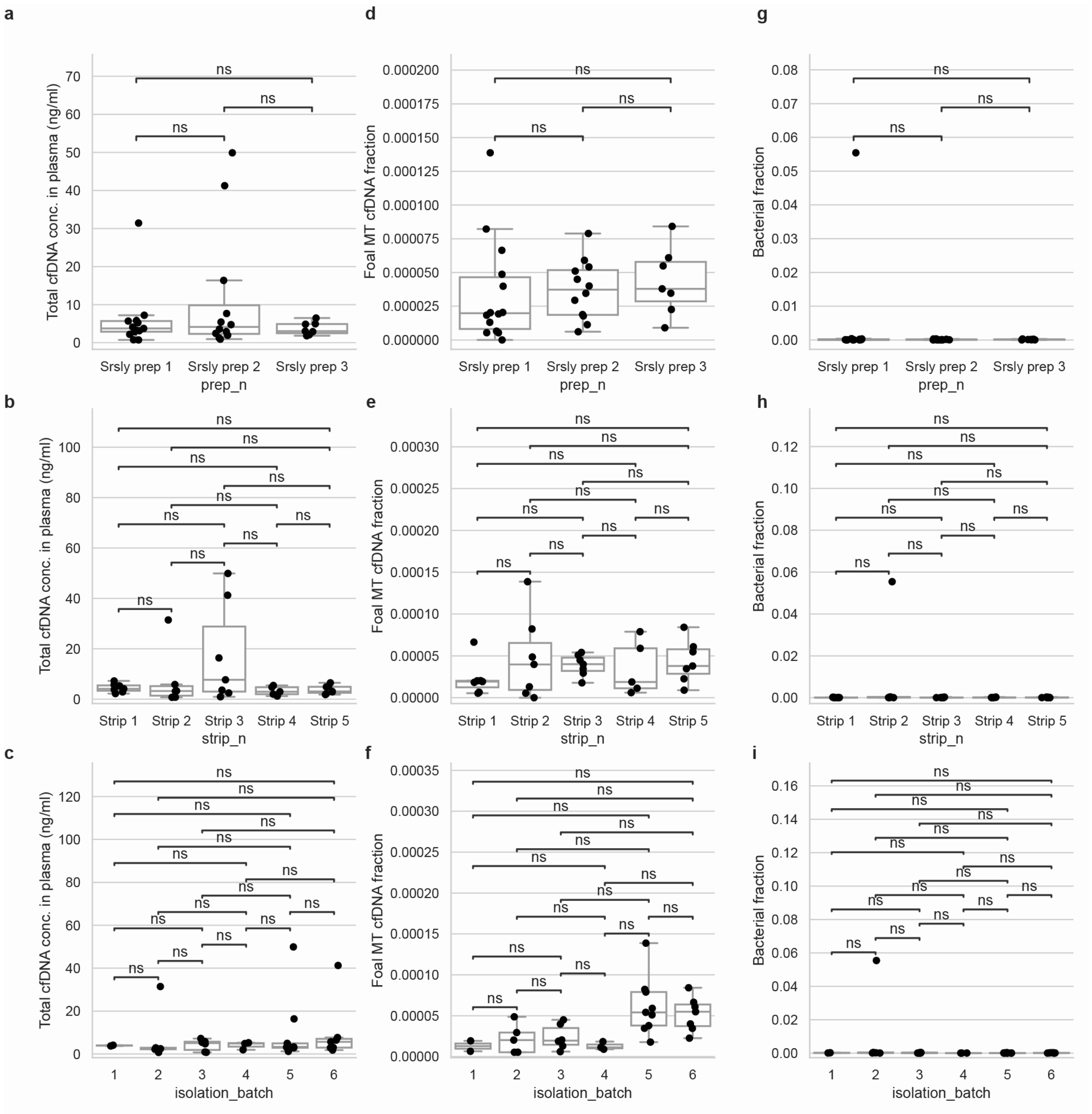

Supplementary Figure S13. No significant batch-associated variation in total plasma cfDNA concentration, foal mitochondrial cfDNA fraction, or bacterial fraction. Related to Figure 4.

Batch effects include different sample preparation methods, strip variations, and isolation batches. Each box plot displays raw data points and represents the 25th percentile (bottom), median (middle), and 75th percentile (top), with whiskers extending to the rest of the distribution within 1.5 times the interquartile range. A Mann-Whitney U Test with Bonferroni correction was performed, revealing no significant differences (ns) among all tests, indicating the robustness and reproducibility of the cfDNA measurement procedures.

a. Total cfDNA concentration in plasma (ng/mL) for three different library preparation batches, SRSLY prep 1, SRSLY prep 2, and SRSLY prep 3.

b. Total cfDNA concentration in plasma (ng/mL) across five different strip locations (Strip 1 to Strip 5).

c. Total cfDNA concentration in plasma (ng/mL) across six isolation batches.

d. Fraction of MT cfDNA for the three library preparation batches.

e. Fraction of MT cfDNA across the five strip locations.

f. Fraction of MT cfDNA across the six isolation batches.

g. Bacterial fraction of cfDNA for the three library preparation batches.

h. Bacterial fraction of cfDNA across the five strip locations.

i. Bacterial fraction of cfDNA across the six isolation batches.

Supplementary Tables S1, S2, S8, and S11.

Clinical characteristics	Age group 1	Age group 2
Age	0-3 days	4-14 days
Temp *	< 37.2 of >39.2 C	< 37.2 of >39.2 C
HR	> 115 /min	>120/min
RR	> 56 /min	> 56 /min
WBC *	<6.9 of >14.4 G/L	<4.0 of > 12.5 G/L
LA	>5 mmol/L	>2.5 mmol/L
Gluc	<2.8 mmol/L	<2.8 mmol/L

* A foal is nSIRS-positive when 3 or more criteria are met, of which one of them is abnormal temp or WBC.

Supplementary Table S1. Age stratified clinical thresholds for nSIRS assessment in foals of age group 1 (0-3 days) and age group 2 (4-14 days). Related to Figure 1a.

The nSIRS-score equals the number of the listed clinical characteristics that meet these specified thresholds. A foal is classified as nSIRS-positive if three or more criteria are met and at least one is an abnormal body temperature or an abnormal white blood cell (WBC) count. nSIRS, neonatal systemic inflammatory response syndrome; Temp, body temperature; HR, heart rate; RR, respiratory rate; WBC, white blood cell count; LA, lactate; Gluc, glucose.

	H	nS-	S+	sS-
**Number of foals (n)**	7	4	11	10
**Admission year**	2021-2022	2021-2022	2021-2022	2021-2022
**Foal age at presentation (hours)**	mean=22, stdev=18	mean=20, stdev=15	mean=26, stdev=28	mean=45, stdev=43
**Gestation length (days)**	missing data	missing data	mean=294, stdev=99	mean=325, stdev=40
**Dam age (years)**	missing data	mean=6, stdev=4	mean=9, stdev=5	mean=10, stdev=4.5
**Dam parity (n)**	missing data	mean=2, stdev=1.5	mean=3, stdev=2.5	mean=3.2, stdev=2.4
**Foal breed**	KWPN*2, Unknown*5	Arabian*1, KWPN*3	Friesian*5, Icelandic horse*1, KWPN*2, Welsh pony*1, Trakehner*1, Unknown*1	KWPN*5, Friesian*2, New Forest* 1, Icelandic horse*1, Unknown*1
**Foal sex**	Mare*1, Stallion*1, Unknown*5	Mare*2, Stallion*2,	Mare*7, Stallion*4	Mare*8, Stallion*2

* if > 50% is missing: 'missing data', no mean and stdev is calculated

Supplementary Table S2. Cohort demographics and perinatal characteristics by clinical group. Related to Figure 1a.

Number of foals (n), admission year, age at presentation (hours), gestation length (days), dam age (years), dam parity (n), foal breed, and foal sex. Continuous variables are summarized as mean and standard deviation (stdev) when ≥50% of values are available; otherwise the field is reported as “missing data.” Breed and sex are shown as counts. stdev, standard deviation; n, number.

Genus	Species	F04	F05	F06	F07	F09	F13	F15	F16	F19	F20	F25
Acinetobacter	*Acinetobacter haemolyticus*	TRUE										
	Acinetobacter lanii										TRUE	
	Acinetobacter wanghuae										TRUE	
Actinobacillus	*Actinobacillus arthritidis*			TRUE							TRUE	
	Actinobacillus equuli		TRUE	TRUE			TRUE	TRUE			TRUE	TRUE
	Actinobacillus genomosp. 1			TRUE							TRUE	
	Actinobacillus genomosp. 2			TRUE								
	Actinobacillus lignieresii			TRUE								
	Actinobacillus pleuropneumoniae			TRUE			TRUE				TRUE	
	Actinobacillus suis			TRUE								
Bacillus	*Bacillus methanolicus*									TRUE		
Enterococcus	*Enterococcus cecorum*									TRUE		
Escherichia	*Escherichia coli*						TRUE				TRUE	TRUE
Pantoea	*Pantoea eucri*		TRUE									
Pseudomonas	*Pseudomonas oryzihabitans*	TRUE										
	Pseudomonas otitidis						TRUE					
	Pseudomonas palleronia											
Staphylococcus	*Staphylococcus equorum*						TRUE					
	Staphylococcus pasteuri		TRUE									
Streptococcus	*Streptococcus equi*								TRUE			
	Streptococcus equinus		TRUE								TRUE	
	Streptococcus suis								TRUE			

Supplementary Table S8. Species-level pathogen detection within 16 sepsis-associated genera in septic foals (S+).

Assessment of species-level elevation in species within 16 genera related to foal sepsis. 22 pathogenic species was detected to be elevated in one or more S+ foals. Columns denote individual foals; marked cells indicate species elevated in that foal.

Oligo name	Oligo sequence
50bp oligo	NNNNNNNCGACACGGATATTCCATCAAGAGACGGGCCTATGGTCCCTGTG ATGATGTNNNNNNN
100bp oligo	NNNNNNNGTAAATCCCACACAGCTGTCGGCTTATATGGTCATTGGACGGC GTAATAGACAAGAGGAGCATCCGTATTACCGCCTATATCGCCTACGTTTAG AGCATTNNNNNNN
150bp oligo	NNNNNNNGCTCTGGTCAGCCTCTAATGGCTCGTAAGATAGTGCAGCCGCT GGTGATCACTCGATGACCTCGGCTCCCCATTGCTACTACGGCGATTCTTG GAGAGCCAGCTGCGTTCGCTAATGTGAGGACAGTGTAGTATTAGCAAACG ATAAGTCNNNNNNN

Supplementary Table 11. Nucleotide sequences of synthetic single-stranded spike-in oligo controls. Related to STAR Methods.

Supplemental Reference List:

[S1] Davis, Nicole M., Diana M. Proctor, Susan P. Holmes, David A. Relman, and Benjamin J. Callahan. 2018. "Simple Statistical Identification and Removal of Contaminant Sequences in Marker-Gene and Metagenomics Data." *Microbiome* 6 (1): 226.
